# Detecting Introgression in Shallow Phylogenies: How Minor Molecular Clock Deviations Lead to Major Inference Errors

**DOI:** 10.1093/molbev/msaf216

**Published:** 2025-09-10

**Authors:** Xiao-Xu Pang, Jianquan Liu, Da-Yong Zhang

**Affiliations:** College of Ecology, Lanzhou University, Lanzhou 730000, China; Ministry of Education Key Laboratory for Biodiversity Science and Ecological Engineering, College of Life Sciences, Beijing Normal University, Beijing 100875, China; College of Ecology, Lanzhou University, Lanzhou 730000, China; Ministry of Education Key Laboratory for Biodiversity Science and Ecological Engineering, College of Life Sciences, Beijing Normal University, Beijing 100875, China

**Keywords:** *D*-statistic, HyDe, false positive, introgression, phylogenomics, rate variation, shallow phylogenies

## Abstract

Recent theoretical and algorithmic advances in introgression detection, coupled with the growing availability of genome-scale data, have highlighted the widespread occurrence of interspecific gene flow across the tree of life. However, current methods largely depend on the molecular clock assumption—a questionable premise given empirical evidence of substitution rate variation across lineages. While such rate heterogeneity is known to compromise gene flow detection among divergent lineages, its impact on closely related taxa at shallow evolutionary timescales remains poorly understood, likely because these taxa are often assumed to adhere to a molecular clock. To address this gap, we combine theoretical analyses and simulations to evaluate the robustness of widely used site pattern methods (*D*-statistic and HyDe) to rate variation across phylogenetic timescales. Our results demonstrate that both methods exhibit high sensitivity to even minor deviations from the molecular clock at shallow timescales, complementing previous findings at deeper scales. Specifically, in young phylogenies (with an age of 3 × 10^5^ generations) with small population sizes, weak (17% difference) and moderate (33% difference) rate variation can inflate false-positive rates up to 35% and 100%, respectively, using site pattern counts from a 500 Mb genome. Employing a more distant outgroup intensifies these spurious signals. Our study demonstrates that summary tests for introgression are pervasively vulnerable to minor rate variations and underscores the critical need for advanced methodologies to disentangle genuine introgression from false signals generated by rate heterogeneity.

## Introduction

Mounting genomic evidence indicates that interspecific gene flow occurs far more extensively than previously recognized across the tree of life (Mallet et al. 2016; Suarez-Gonzalez et al. 2018; Taylor and Larson 2019; Edelman and Mallet 2021). As Edelman and Mallet (2021) (p. 271) observed, “phylogenies with no evidence of gene flow are beginning to seem like the exception rather than the rule.” Testing for gene flow (or introgression) has become a routine component of phylogenetic analyses, enabling researchers to evaluate gene flow's role in a group's species diversification and to guide the selection between tree-based and network-based evolutionary frameworks (Blair and Ané 2020; Haque and Kubatko 2024).

Over the past few years, various introgression detection approaches have been developed within a rigorous hypothesis-testing framework (Jiao et al. 2021; Hibbins and Hahn 2022). These methods can be classified into 2 main categories based on the type of information utilized. The first category relies on gene-tree topologies or parsimony-informative site patterns, detecting gene flow through significant asymmetries between discordant gene trees or between discordant site patterns, as exemplified by the widely used *D*-statistic (Green et al. 2010), HyDe (Blischak et al. 2018), and MSCquartets (Rhodes et al. 2021). The second utilizes gene-tree branch lengths as a source of information, examining whether the branch length distributions deviate from expectations under conditions involving only incomplete lineage sorting (ILS). Examples include *D_3_* (Hahn and Hibbins 2019), QuIBL (Edelman et al. 2019), and the full-likelihood tests that make full use of both topological and branch length information in gene trees (Zhu and Yang 2012; Ji et al. 2023). Particularly, site pattern-based methods operate directly on the whole genome sequence alignment data, thus avoiding potential errors introduced by preprocessing steps such as loci partitioning and gene-tree estimation from individual loci.

Despite significant advances in introgression testing methods, each approach relies, to varying degrees, on various theoretical assumptions, and violations of these assumptions can lead to erroneous conclusions. One such assumption—the molecular clock or rate constancy among lineages, which is either implicitly or explicitly built into many widely used methods—has recently garnered considerable attention (Blair and Ané 2020; Frankel and Ané 2023; Koppetsch et al. 2024). For instance, the *D*-statistic assumes no multiple hits—that is, each site undergoes at most 1 mutation (Durand et al. 2011). Under this assumption, the discordant sites *ABBA* and *BABA* can only arise from a single substitution event along the internal branches of discordant gene trees. Gene flow is then assumed to be the only possible mechanism disrupting the *ABBA–BABA* balance, which is otherwise maintained by ILS. However, these discordant site patterns may also result from homoplasies, where identical alleles arise independently in different lineages. When rate variation exists between sister lineages, homoplasies can create *ABBA–BABA* asymmetry, mimicking introgression. Such false positives in the *D*-statistic have been confirmed in recent simulation studies by Frankel and Ané (2023) and Koppetsch et al. (2024), which examined cases involving deep divergences where sister lineages have been separated for over a million generations. However, it remains unclear whether this same caveat applies to shallow phylogenies, where only moderate deviations from the clock assumption are possible at most. Additionally, a significant limitation of these studies is their reliance solely on simulations, without theoretical analysis to support their findings.

**Fig. 1.**
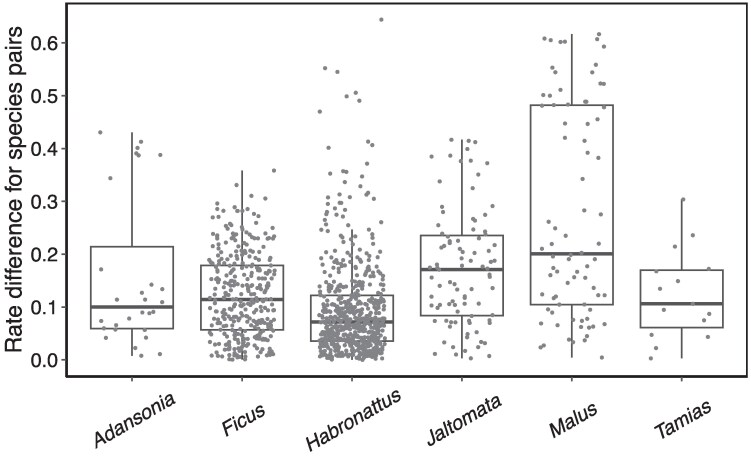
Evolutionary rate differences among species pairs across 6 genera, calculated using the relative rate test (see Graur and Li 2000, supplementary note S1, Supplementary Material online). For a genus with *n* species, all *n*(*n*  *−* 1)/2 intra-generic species pairs were analyzed. Each point represents the rate difference between one such pair, defined as the difference in lengths between 2 branches divided by the length of the longer branch.

Substitution rates are shaped by various factors such as generation time, effective population size, metabolic rate, and longevity (Bromham 2009; Thomas et al. 2010; Lanfear et al. 2014). Since some of these factors can vary considerably over short timescales, substitution rates may differ significantly even among closely related species, as evidenced by numerous empirical studies (Bromham et al. 2013; Dann et al. 2017; Saclier et al. 2018; Bergeron et al. 2023). To better understand the prevalence and magnitude of rate variation within shallow phylogenies, we employed the relative rate test (Graur and Li 2000) to quantify rate differences between a pair of lineages and applied it to published datasets from a random selection of 6 genera (see supplementary note S1, Supplementary Material online), spanning both plant and animal taxa (Leduc-Robert and Maddison 2018; Wu et al. 2018; Karimi et al. 2020; Sarver et al. 2021; Liu et al. 2022; Gardner et al. 2023). Notably, intra-generic species frequently exhibit rate disparities of 10% to 30%, with some species pairs in *Habronattus* and *Malus* even exceeding 50% (Fig. 1). Thus, rate variation among closely related species is both widespread and appreciable, underscoring the need to account for such variation in evolutionary analyses. Moreover, the *D*-statistic was originally designed for testing hybridization between humans and Neanderthals, which diverged about 20,000 generations ago (Green et al. 2010), and remains predominantly applied to groups with comparable evolutionary scales (i.e. within tens of thousands to a few millions of generations). Given the widespread use of the *D*-statistic and other site pattern-based methods in shallow phylogenies, it is critical to assess and quantify their reliability in the presence of rate variation across lineages.

Our study aims to assess and quantify the robustness of the site pattern-based *D*-statistic and HyDe methods against lineage-specific rate variation at shallow phylogenetic scales. We began with a mathematical analysis to derive the expected *D*-values under varying degrees of rate variation between sister lineages, along with other key parameters such as phylogenetic age, effective population size, and outgroup distance. We then simulated a range of scenarios spanning phylogenetic depths of 10^4^ to 10^6^ generations to examine the behaviors of the *D*-statistic and HyDe in the presence of rate variation. Our results reveal the widespread conditions under which rate variation generates false-positive signals of introgression in popular summary tests, calling into question the validity of numerous reported gene flow events, which may, in fact, be artifacts of substitution rate heterogeneity.

## Theory

We begin with an introduction to the basic principles of the site pattern-based methods *D*-statistic (Green et al. 2010) and HyDe (Blischak et al. 2018). In the 4-taxon asymmetric species tree *S* with the topology of (((*P1*, *P2*), *P3*), *O*), when introgression is absent, 2 discordant unrooted gene trees shaped by ILS, *p1p3|p2o* and *p2p3|p1o*, occur with equal probabilities but at lower frequencies than *p1p2|p3o* (Pamilo and Nei 1988; Degnan 2013). Building on this property, *D*-statistic and HyDe assume no multiple hits and use parsimony-informative site patterns—*ABBA*, *BABA*, and *BBAA* (with *A* representing the reference state from the outgroup listed last)—as proxies for 3 gene-tree topologies. Introgression is detected by identifying deviations from the expected symmetry between *ABBA* and *BABA* counts. Specifically, the *D*-statistic, calculated as:


$$D=\frac{N_{ABBA}-N_{BABA}}{N_{ABBA}+N_{BABA}},$$

detects introgression when the *D*-value significantly deviates from 0. When D>0, indicating an excess of *ABBA* over *BABA*, the *D*-statistic suggests introgression between *P2* and *P3*; conversely, when D<0, it points to introgression between *P1* and *P3*. HyDe employs a different statistic (refer to Kubatko and Chifman 2019 for further details) but fundamentally tests whether the 2 least frequent site patterns occur at comparable frequencies. A significant result is interpreted as indicative of a hybrid speciation event, where the 2 parental species are more distantly related to each other than to the hybrid species. Thus, the 2 ingroup lineages that share alleles in the smallest number of parsimony-informative sites will be identified as putative parents, with the remaining lineage considered the hybrid (Kubatko and Chifman 2019). It should be noted that, while ghost introgression can also yield significant *D*-values (Tricou et al. 2022; Pang and Zhang 2024), here we do not incorporate ghost lineages into consideration for the sake of conceptual simplicity.

**Fig. 2.**
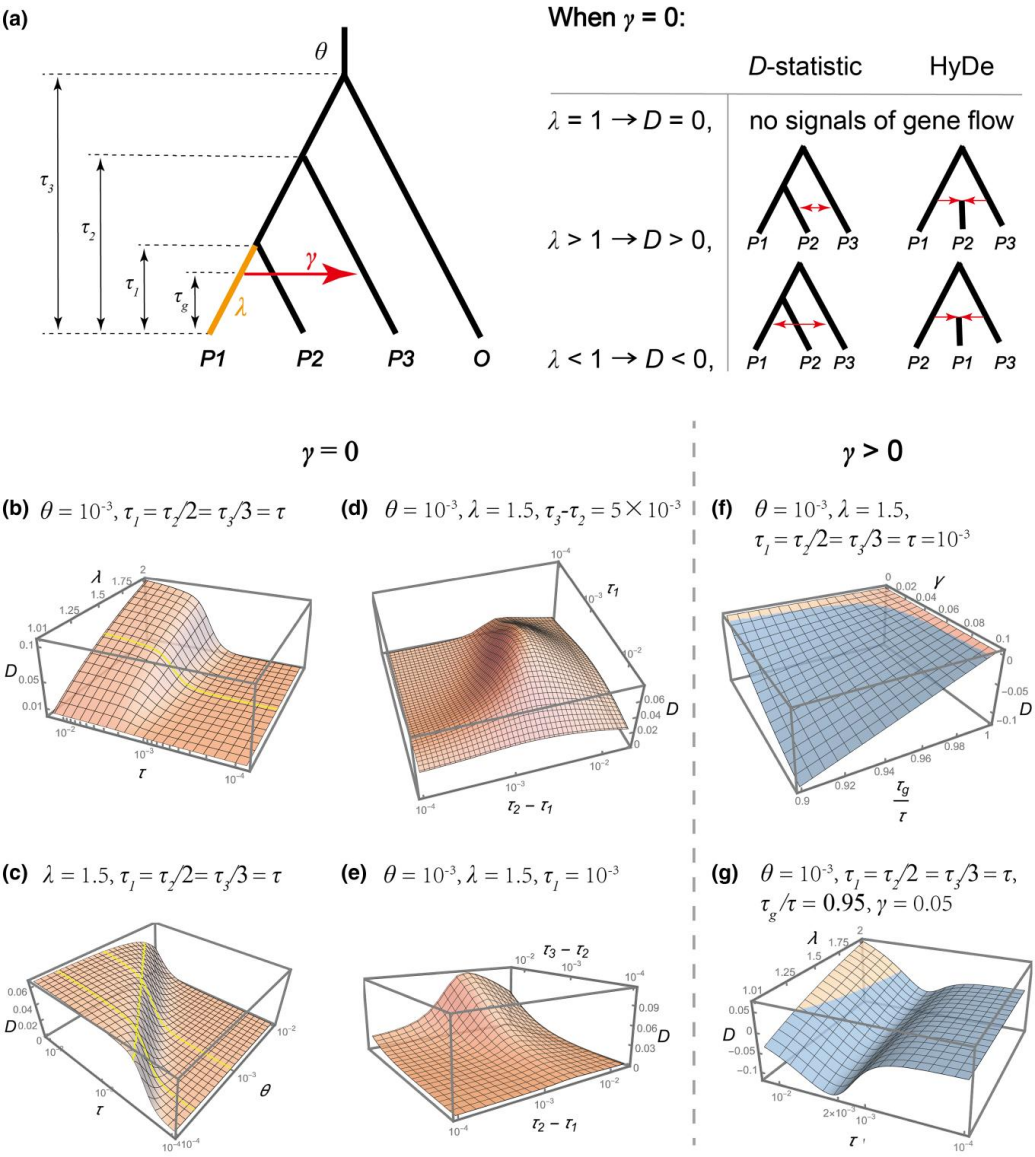
Impact of rate variation on introgression testing. a) Left panel: a 4-taxon asymmetric species tree with an outflow introgression (*P1* → *P3*) and rate variation between sister lineages. The introgression probability is denoted as *γ*. Divergence time $\tau_{i}\;\{i=1,\;2,\;3\}$, introgression time $\tau_{g}$, and the ancestral population size *θ* are measured in terms of the expected number of mutations per site. The lineage *P1*, highlighted in orange, exhibits a distinct substitution rate relative to other lineages, with *λ* representing its relative rate. Right panel: expected behaviors of the *D*-statistic and HyDe under different conditions of *λ* in the absence of introgression (γ=0). b to e) Plots illustrating the effect of various parameters on *D*-values in the absence of introgression (γ=0). Axis for *τ* and *θ* are log-scaled. Note that in some panels, the orientation of the horizontal axes is reversed (e.g. *τ* increases from right to left in panel b) to enhance visualization of surface trends. The yellow curve in panel b marks λ=1.5. Yellow curves in panel c correspond to $\theta =2\times 10^{-4}$, $\theta =10^{-3}$, and D=0.05. f and g) Plots illustrating the effect of various parameters on *D*-values in the presence of introgression (γ>0). Orange and blue surfaces in 3D plots indicate parameter regions where *D* > 0 and *D* < 0, respectively.

In our previous study (Pang and Zhang 2024), the frequencies of 3 parsimony-informative site patterns are derived under the infinite-site model. Here, we relax this assumption to allow for homoplasies and further incorporate rate variation between sister lineages. We use the multispecies coalescent with introgression (MSci; Flouri et al. 2020) model to consider an episodic introgression event from P1 to P3 within the species tree *S*, with the introgression proportion denoted by *γ* (Fig. 2a). Speciation and introgression times, τ=Tμ, and population sizes, θ=4Nμ, are measured in terms of the expected number of mutations per site, where *T* is the time in generations, *N* is the number of diploid individuals, assumed constant across lineages, and *μ* represents the mutation rate per site per generation. To account for rate variation between a pair of sister lineages, we introduce a relative rate parameter *λ* for lineage *P1*, scaling its branch length to $\lambda \tau_{1}$ and population size to λθ accordingly. Gene-tree distributions are calculated under the MSci model, and sequence evolution along gene trees is modeled using the Jukes–Cantor substitution model (JC69; Jukes and Cantor 1969) to accommodate multiple substitutions at the same site. The frequencies of 3 site patterns per nucleotide site are derived by traversing all possible gene-tree histories and are provided in supplementary note S2, Supplementary Material online.

### Case 1: γ=0 (No Introgression)

We first consider the case where γ=0. The expected frequency difference between *ABBA* and *BABA* is given by:


(1) $$\begin{array}{c} P(ABBA)-P(BABA)=\frac{3}{16}\frac{1-e^{-\frac{4}{3}(\lambda -1)\tau_{1}}}{\left(1+4\theta /3\right)}\left(e^{-\frac{8\tau_{2}}{3}}-e^{-\frac{8\tau_{3}}{3}}\right) \end{array}$$

where $\tau_{3}>\tau_{2}>\tau_{1},\tau_{i},\;\lambda \in (0,\infty )$. It follows that the relative magnitudes of ABBA and *BABA* counts are determined by the parameter *λ*. Additionally, the inequality P(BBAA)>P(BABA) necessarily holds, as demonstrated in supplementary note S3, Supplementary Material online. From these observations, we can draw the following conclusions:


(2) $$\begin{array}{c} \mathrm{if}\;\lambda =1,\;P(BBAA)>P(ABBA)=P(BABA)\\ \mathrm{if}\;\lambda >1,\;min\{P(BBAA),P(ABBA)\}>P(BABA)\\ \mathrm{if}\;\lambda <1,P(BBAA)>P(BABA)>P(ABBA) \end{array}$$

When λ=1 (i.e. no rate variation), the *ABBA–BABA* balance is maintained (D=0) even in the presence of homoplasies. However, when λ≠1, this balance is disrupted. Specifically, when λ>1, indicating an accelerated rate for *P1* lineage, D>0, *D*-statistic interprets as introgression between lineages *P2* and *P3*, while HyDe infers *P1* and *P3* as the parental lineages—those sharing alleles in the fewest site pattern *BABA*—and *P2* as the hybrid lineage (Fig. 2a). Conversely, when λ<1, reflecting a slower rate of *P1*, there is D<0, *D*-statistic interprets as introgression between *P1* and *P3*, while HyDe would classify *P2* and *P3* as the parental lineages—those sharing the derived state in the fewest site pattern *ABBA*—and *P1* as the hybrid lineage (Fig. 2a).

Quantitatively, the absolute difference between *ABBA* and *BABA* primarily depends on the terms $1-e^{-4(\lambda -1)\tau_{1}/3}$ and $e^{-8\tau_{2}/3}-e^{-8\tau_{3}/3}$ in Equation (1), which reflect the length differences between sister lineages *P1* and *P2*, and between *P3* and the outgroup *O*, respectively. Obviously, greater length differences augment the extent of *ABBA–BABA* asymmetry. Consider the extreme cases where *P1* and *O* exhibit long branches while *P2* and *P3* have negligible ones (i.e. $\tau_{1},\tau_{2}\to 0$, and $\lambda \tau_{1},\tau_{3}\to \infty$), the majority of homoplasies occur along the *P1* and *O* lineages to generate *ABBA* sites. Consequently, *D*-value approaches 1 if θ≪1 (detailed proof in supplementary note S4, Supplementary Material online). Conversely, if the *P1–P2* or *P3–O* pair has equal branch lengths (i.e. λ=1 or $\tau_{2}=\tau_{3}$), *D* equals 0.

Next, we analyze the effects of various parameters on the *D*-value. First, we plot the *D* against *λ* and *τ*, assuming $\tau_{1}=\tau_{2}/2=\tau_{3}/3=\tau$ and fixing $\theta =10^{-3}$ (Fig. 2b). The results indicate that *D* generally increases with *λ* and *τ*, in line with the expectation that greater rate variation and deeper phylogeny amplify spurious introgression signals. Specifically, *D* shows an approximately linear increase with *λ*, while following an S-shaped curve with respect to *τ*, featuring a sharp rise around $\tau =10^{-3}$ and leveling off near $\tau =3\times 10^{-3}$. For example, with λ=1.5 (Fig. 2b), increasing *τ* from $10^{-3}$ to $3\times 10^{-3}$ results in a pronounced rise in *D* from 0.006 to 0.055; further increasing *τ* to $10^{-2}$ only causes a slight increase in *D*, reaching a value of 0.063.

Then, we plot the *D* against *τ* and *θ*, fixing λ=1.5 (Fig. 2c). The results reveal a strong interaction between *θ* and *τ* in determining *D*-values, where smaller *θ* values necessitate lower phylogenetic depth (*τ*) thresholds for a high *D*-value. For instance, when $\theta =10^{-3}$, a *τ* value of $2.4\times 10^{-3}$ is required to reach *D* = 0.05, whereas for $\;\theta =2\times 10^{-4}$, only $5.8\times 10^{-4}$ of *τ* is needed (Fig. 2c). This pattern arises because larger *θ* amplifies ILS, which contributes equally to *ABBA* and *BABA*, thereby diluting their difference caused by homoplasies.

Finally, we evaluate the effects of individual branch lengths, specifically $\tau_{1}$, $\tau_{2}-\tau_{1}$, and $\tau_{3}-\tau_{2}$, while fixing $\theta =10^{-3}$ and λ=1.5. When *D* is plotted against $\tau_{1}$ and $\tau_{2}-\tau_{1}$, with $\tau_{3}-\tau_{2}=5\times 10^{-3}$ fixed, it exhibits a unimodal pattern for both variables, initially increasing and then decreasing (Fig. 2d). The initial rises with $\tau_{1}$ and $\tau_{2}-\tau_{1}$ are driven by an accumulation of homoplasy-induced sites and a reduction in ILS-induced sites, respectively (supplementary fig. S3, Supplementary Material online). The subsequent decline in *D*, though counterintuitive, can be explained by Equation (1): Increasing both variables elongates the branch lengths of *P3* and *O* (i.e. $\tau_{2}$ and $\tau_{3}$; note that the increase in $\tau_{3}$ results from increasing $\tau_{2}$ while keeping $\tau_{3}-\tau_{2}$ constant), thereby reducing the value of the term $e^{-8\tau_{2}/3}-e^{-8\tau_{3}/3}$ and diminishing the *ABBA–BABA* difference, despite an increased occurrence of homoplasies. When *D* is plotted against the outgroup distance $\tau_{3}-\tau_{2}$ with $\tau_{1}=10^{-3}$ fixed, *D* increases with larger values of $\tau_{3}-\tau_{2}$ (Fig. 2e). This effect is driven by increasing branch length disparities between *P3* and the outgroup *O*, which intensify homoplasy-induced asymmetry.

### Case 2: γ>0 (Introgression Present)

We next examine the cases where γ>0, indicating the presence of gene flow. First, we plot the *D* against *γ* and $\tau_{g}/\tau$, with $\;\tau =\tau_{1}=\tau_{2}/2=\tau_{3}/3=10^{-3},$  $\theta =10^{-3}$, and λ=1.5 fixed (Fig. 2f). The results show that both the degree and timing of introgression, *γ* and $\tau_{g}/\tau$, have a significant impact on *D*-values. More intense and recent introgression strengthens introgression signals, making them less susceptible to rate variation. Only in a limited parameter space, where γ<0.03 or $\tau_{g}/\tau >0.98$, does rate variation result in *D* close to 0 or even positive (as indicated by the orange region of Fig. 2f).

We then fix $\tau_{g}/\tau =0.95$ and γ=0.05 and plot *D* against *λ* and *τ* to explore the effect of phylogenetic age (Fig. 2g). When λ=1, as expected, D<0. Interestingly, the absolute value of *D* follows a nonmonotonic pattern, initially increasing and then decreasing as *τ* increases. This may suggest that introgression signals are strongest at moderate phylogenetic depths. Further increases in *λ* lead to a rise in *D*, and for larger *τ*, the sign of *D* even reverses from negative to positive (Fig. 2g).

## Simulations

**Fig. 3.**
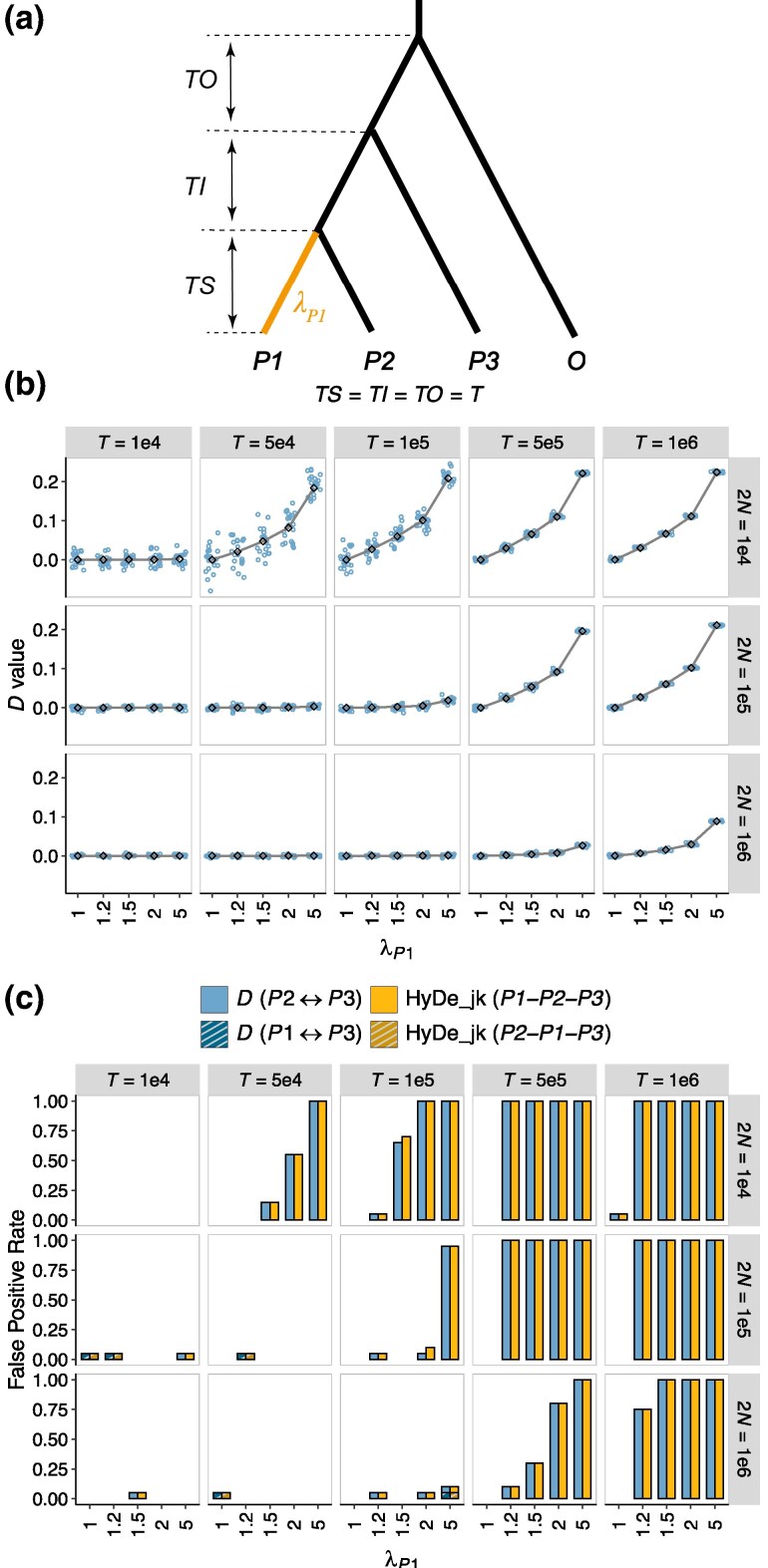
Interactive effects of phylogenetic depth (*T*), population size (2*N*), and rate variation on summary tests for introgression. a) Simulated demographic histories. Branch lengths (*TS*, *TI*, and *TO*) are measured in generations. $\lambda_{P1}$ represents the relative rate of the lineage *P1*. b and c) Results of introgression tests under the condition where *TS* = *TI* = *TO* = *T*. The values on the strips at the top and right of each plot indicate the phylogenetic depth (*T*) and population size (2*N*), respectively. $\lambda_{P1}$ is labeled on the x-axis. b) *D*-values: colored points represent *D* estimates, with solid lines indicating the theoretical expected *D*. c) False-positive rate of the *D*-statistic and HyDe_jk. “*Pi* ↔ *Pj*” represents the inference of introgression between lineages *Pi* and *Pj* in the *D*-statistic; “*Pi-Pj-Pk*” indicates the identification of *Pj* as the hybrid lineage, with *Pi* and *Pk* acting as the parental lineages in HyDe_jk, where HyDe_jk refers to HyDe with a block-jackknife procedure for *P*-value calculation.

To assess the robustness of introgression testing methods to rate variation among lineages, we simulated 8 different scenarios (Table 1), each with distinct focus points. Simulations were conducted under the species tree ***S*** (Fig. 3a), either without introgression (Scenarios 1 to 7) or with introgression (between *P1* and *P3*; Scenario 8). Time parameters (*TS*, *TI*, *TO*, and *TG*) were measured in generations. The parameters $\lambda_{P1},$  $\lambda_{P3}$, and $\lambda_{O}$ represent the relative rates of *P1*, *P3*, and *O*, compared to the original rate at the root. To compare the quantitative effects of rate acceleration and deceleration, we defined the rate difference as the absolute difference between the original and altered rates, normalized by the faster of the two. Thus, both λ=x(x>1) and λ=1/x correspond to a rate difference of 1−1/x. We examined 4 relative rate pairs: 0.2 vs. 5, 0.5 vs. 2, 0.67 vs. 1.5, and 0.83 vs. 1.2, with λ=1 serving as the baseline. Detailed parameter configurations for each scenario are provided in Table 1.

**Table 1:** The simulation scenarios and parameter settings

Scenario	*TS*	*TI*	*TO*	2*N*	$\lambda_{P1}$	$\lambda_{P3}$	$\lambda_{O}$	GS
1 **(**Fig. 3, supplementary fig. S5, Supplementary Material online**)**	1e4, 5e4, 1e5, 5e5, 1e6	*TI* = *TS*	*TO* = *TS*	1e4, 1e5, 1e6	0.2, 0.5, 0.67, 0.83, 1, 1.2, 1.5, 2, 5	1	1	100 M
2 **(**Fig. 4)	1e4, 5e4, 1e5, 5e5, 1e6	1e5	*TO* = *TI*	1e4	1.5	1	1	100 M
3 (Fig. 4)	1e5	1e4, 5e4, 1e5, 5e5, 1e6	*TO* = *TS*	1e4	1.5	1	1	100 M
4 (Fig. 4)	1e5	*TI* = *TS*	1e4, 5e4, 1e5, 5e5, 1e6	1e4	1.5	1	1	100 M
5 (Fig. 5)	1e5	*TI* = *TS*	*TO* = *TS*	1e4	1, 1.5	0.5, 0.67, 1, 1.5, 2	1	100 M
6 (Fig. 5)	1e5	*TI* = *TS*	*TO* = *TS*	1e4	1, 1.5	1	0.5, 0.67, 1, 1.5, 2	100 M
7 (Fig. 6)	1e5	*TI* = *TS*	*TO* = *TS*	1e4	1.2, 1.5	1	1	100 M, 500 M, 1 G
8 γ=0.05 ***TG*** **=** **0.95*TS*** (Fig. 7, supplementary fig. S7, Supplementary Material online)	1e4, 5e4, 1e5, 5e5, 1e6	*TI* = *TS*	*TO* = *TS*	1e4, 1e5, 1e6	0.2, 0.5, 0.67, 0.83, 1, 1.2, 1.5, 2, 5	1	1	100 M

*TS*: Divergence Time of Sister Lineages; *TI*: Internal Branch Length; *TO*: Distance of Outgroup; 2*N*: Effective Population Size; *λ*_*i*_: Relative Rate of the branch *i*; *GS*: Genome Size.

For each scenario, we first used fastsimcoal v2.8 (Excoffier et al. 2013) to simulate gene trees, with a single sample per lineage. Since fastsimcoal2 does not natively support evolutionary rate variation among lineages, we accounted for this by scaling branch lengths by *λ* through controlling sampling times of samples. For example, if the evolutionary rate of the *P1* lineage was accelerated ($\lambda_{P1}>1$), all divergence times were shifted into the past by $(\lambda_{P1}-1)*TS$, with *P1* sampled at the present, while all other extant lineages were sampled at $(\lambda_{P1}-1)*TS$ generations ago. We then simulated 100-bp-long sequences from each gene tree using Seq-Gen software (Rambaut and Grass 1997) under the JC69 model and a mutation rate $\mu =2\times 10^{-8}$ per site per generation (“-s 2e-8”). The per-gene sequences were concatenated to generate genome sizes of 100 Mb, unless otherwise specified. To examine the impact of recombination levels on introgression testing, we also generated additional datasets under Scenario 1, with locus length increased to 1,000 bp while keeping the total genome size. Each parameter combination was simulated with 20 replications.

Both the *D*-statistic and HyDe methods were applied to each dataset. HyDe v.0.4.3 (Blischak et al. 2018) was run using the “run_hyde.py” script, with *O* designated as the outgroup. To account for site dependence, we implemented a block-jackknife approach for HyDe via a custom Python script and refer to this modified version as “HyDe_jk.” For the *D*-statistic, we calculated *D*-values based on site pattern counts provided by HyDe and estimated the standard error *SE*(*D*) using the block-jackknife method. The significance of introgression was assessed via z=D/SE(D), with a *P*-value threshold of 0.01.

## Results

### Effect of Phylogeny Age and Population Size

In this section, we simulated the scenarios without introgression (Fig. 3a, parameters: Scenario 1 in Table 1). Assuming branch lengths *TS*  *=*  *TI*  *=*  *TO*  *=*  *T*, we varied the parameter *T* and 2*N* to investigate how phylogenetic depth interacts with population size to influence the occurrence of false positives caused by rate variation. The results are summarized in Fig. 3 and supplementary fig. S5, Supplementary Material online.

In the absence of rate variation ($\lambda_{P1}=1$), *D*-values, as expected, remained close to 0, and the FP rates of the *D*-statistic and HyDe were low across all parameter combinations. When the evolutionary rate of the *P1* lineage accelerated ($\lambda_{P1}>1$), *D*-values increased and became positive (Fig. 3b). Under these conditions, the *D*-statistic detected significant signals of introgression between the *P2* and *P3* lineages in many parameter combinations, and HyDe misidentified the *P2* lineage as the hybrid (Fig. 3c). On the other hand, when the evolutionary rate of the *P1* lineage slowed ($\lambda_{P1}<1$), *D*-values turned negative, suggesting introgression between the *P1* and *P3* lineages. In this case, HyDe misidentified the *P1* lineage as the hybrid (supplementary fig. S5, Supplementary Material online). These results align with our theoretical predictions illustrated in Fig. 2a. Notably, we observed that for the same degree of rate variation (i.e. $\lambda_{P1}=k$ versus $\lambda_{P1}=1/k$), a decrease in the evolutionary rate of *P1* leads to FP rates similar to those observed when the rate is accelerated, though slightly lower.

We found that *D*-values deviated further from 0, and FP rates increased with greater phylogenetic depth (*T*) and smaller population sizes (2*N*). For example, when 2*N* = $10^{4}$ and *T* = $10^{5}$, a 50% increase in substitution rate ($\lambda_{P1}=1.5$, corresponding to 33% rate difference) resulted in a mean *D*-value of 0.063 (range: 0.0279 to 0.1097) and approximately 70% FP rate (Figs. 3b and c). In contrast, with a larger population size of 2*N* = $10^{5}$, the mean *D*-value dropped to around 0 (range: −0.01304 to 0.0117), and the FP rate became negligible.

We further examined the performances of the *D*-statistic and HyDe when the locus length was increased to 1,000 bp while holding the total genome size constant at 100 Mb, for the parameter combinations with *T* ∈ {10^5^, 5 × 10^5^}, 2*N* ∈ {10^4^, 10^5^}, and $\lambda_{P1}$ ∈ {1.2, 1.5, 2}. The corresponding results were presented in supplementary fig. S6, Supplementary Material online. For all combinations, both 2 methods showed comparable FP rates with those observed with 100 bp datasets (supplementary fig. S6, Supplementary Material online). This consistency reflects the limited impact of increased locus length (or recombination level) under shallow divergence scenarios, where the rarity of mutations leads to sparse SNP distribution across the genome, maintaining their independence even over longer linked regions.

### Effects of Divergence Time of Sister Lineages, Internal Branch Length, and Distance of Outgroup

**Fig. 4.**
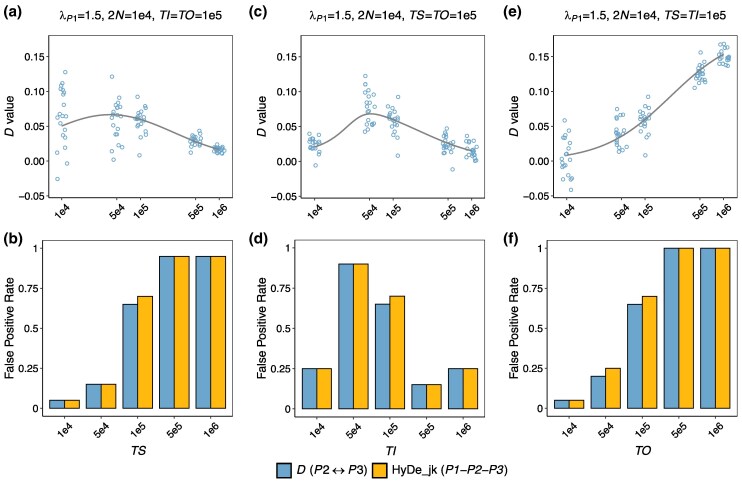
Interactive effects of individual branch lengths (*TS*, *TI*, and *TO*) and rate variation on summary tests for introgression. The demographic scenario corresponds to Fig. 3a, with $\lambda_{P1}=1.5$ and $2N=10^{4}$. Default branch lengths are set to *TS* = *TI* = *TO* = 10^5^ generations. a and b) *D*-values and false-positive rates with varying *TS*. Colored points in panel a represent *D*-value estimates, with solid lines indicating the theoretically expected *D*-values. c and d) Results with varying *TI*. e and f) Results with varying *TO*. See Fig. 3 for an explanation of the symbol legends.

We further investigated the effects of individual branch lengths *TS*, *TI*, and *TO*. In these analyses, we considered scenarios (Scenarios 2 to 4 in Table 1) with a rate variation $\lambda_{P1}=1.5$ and a population size of 2*N* = 10^4^. We initialized *TS* = *TI* = *TO* = 10^5^ generations and independently varied each branch length across a range of 10^4^ to 10^6^ generations to assess its specific effects on introgression testing outcomes. The results are displayed in Fig. 4.

First, we examined the effect of the divergence time of sister lineages *TS*. The *D*-values initially increased with *TS* but showed a decline when *TS* > 5 × 10^4^ (Fig. 4a). This later decline trend was also observed in a study by Koppetsch et al. (2024) in the context of deep divergence of sister lineages (>10 million generations). Interestingly, the FP rates exhibited a different trend, steadily increasing as *TS* increased (Fig. 4b). FP rates were approximately 15% at *TS* = 5 × 10^4^ and reached nearly 100% when *TS* = 10^6^, even though the mean *D*-value was obviously higher at *TS* = 5 × 10^4^ (0.059) compared to *TS* = 10^6^ (0.017). This discrepancy may arise because larger *TS* values contribute more homoplasy-induced *ABBA* and *BABA* sites, reducing sample variance, as reflected by the lower variance of *D*-values across replicates. This, in turn, results in a higher z-score, despite the slight decline in *D*-values. Next, for the internal branch length *TI*, both *D*-values and FP rates followed a nonmonotonic trend: initially rising with *TI* and then decreasing, peaking at *TI* = 5 × 10^4^ with a mean *D*-value of 0.078 and FP rate of approximately 75% (Fig. 4c and d). Finally, for the outgroup distance *TO*, *D*-values gradually deviated from 0 with increasing *TO*, reaching a striking value of ∼0.15 at $TO=10^{6}$ (Fig. 4e). FP rates followed a similar trend, rising from nearly 0% at $TO=10^{4}$ to ∼70% at $TO=10^{5}$ and reaching 100% at $TO\geq 5\times 10^{5}$ (Fig. 4f). This suggests that a larger distance between the outgroup and the ingroups exacerbates the likelihood of false positives due to rate variation.

### Effects of P3- or O-mutation Rate Variation

**Fig. 5.**
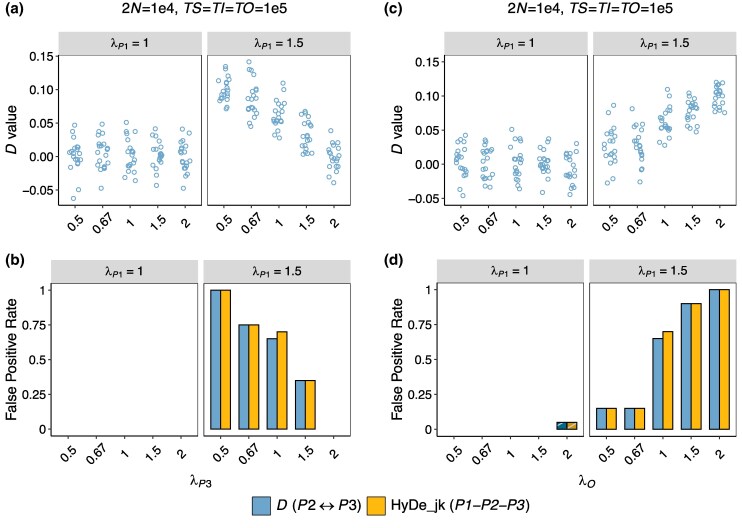
Effects of *P3*- and *O*-rate variation on summary tests for introgression. The simulated demographic scenario corresponds to Fig. 3a, with additional rate variation in lineages *P3* and *O*, where $\lambda_{P3}$ and $\lambda_{O}$ denote their relative rates. Branch lengths are fixed at *TS* = *TI* = *TO* = 10^5^ generations, and $2N=10^{4}$. a and b) *D*-values and false-positive rates with varying $\lambda_{P3}$. The top strips of each plot indicate conditions for $\lambda_{P1}=1$ and $\lambda_{P1}=1.5$, corresponding to scenarios without and with rate variation between sister lineages, respectively. Colored points in panel a represent *D*-value estimates. c and d) Results with varying $\lambda_{O}$. See Fig. 3 for an explanation of the symbol legends.

This section extends the analysis to the effects of rate variation in *P3* and the outgroup *O* on introgression testing. We considered the following scenarios (Scenarios 5 to 6 in Table 1) with branch lengths of *TS* = *TI* = *TO* = 10^5^ generations and a population size of 2*N* = 10^4^. The relative rates of *P3* and *O*, $\lambda_{P3}$ and $\lambda_{O}$, were varied under conditions with and without rate variation between sister lineages. The corresponding results are presented in Fig. 5.

In the absence of rate variation between sister lineages (i.e. $\lambda_{P1}=1$), the estimated *D*-values remained consistently around 0 regardless of changes in the rates of *P3* or *O*. In this case, all methods performed well, with the rejection rates of the *D*-statistic and HyDe at 1% and 1.5%, respectively (Fig. 5).

When rate variation was present among sister lineages (i.e. $\lambda_{P1}=1.5$), reducing the *P3*-mutation rate led to a noticeable increase in both *D*-values and FP rates (Fig. 5a and b). For instance, a 50% reduction in the *P3*-mutation rate (i.e. $\lambda_{P3}=0.5$) increased the mean *D*-value from 0.063 to 0.1 and the FP rate from ∼70% to 100%. Conversely, doubling the *P3*-rate (i.e. $\lambda_{P3}=2$) reduced the mean *D*-value to nearly 0 and eliminated false positives. The effects of rate variation in the outgroup *O* followed an opposite trend (Fig. 5c and d). Halving the *O*-rate ($\lambda_{O}=0.5$) decreased the mean *D*-value to 0.027 and the FP rate to 15%, while doubling the *O*-rate ($\lambda_{O}=2$) increased the mean *D*-value to 0.1 and the FP rate to 100%.

### Effects of Genome Size (or Amount of Data)

In statistical testing, larger sample sizes generally enhance statistical power. Given the wide variation in genome sizes across animals and plants, ranging from 12 Mb to 27.6 Gb, with a median of 517 Mb and a mean of 1.21 Gb (Kress et al. 2022), we extended our analysis beyond the initial 100 Mb dataset to explore the impact of larger genome sizes. Here, in Scenario 7 (Table 1) with branch lengths of *TS* = *TI* = *TO = T* ranging from 10^4^ to 10^5^ generations, rate variation of $\lambda_{P1}=1.2$ or 1.5, and a population size of 2*N* = 10^4^, we simulated larger genome sizes of 500 Mb and 1 Gb to explore the effects of genome size on the rate variation-induced FP rates.

The results show that, as expected, the estimated *D*-values across replicates become less scattered as genome size increases, converging toward the theoretically expected value (indicated by the gray lines). Meanwhile, the FP rates of both methods rise significantly with genome size. Under weak rate variation of $\lambda_{P1}=1.2$ and *T* = 10^5^ generations, expanding the dataset size from 100 to 500 Mb and 1 Gb elevated the FP rate from 5% to approximately 35% and 60%, respectively. Under stronger rate variation ($\lambda_{P1}=1.5$), FP rates reached ∼100% at just 500 Mb. These results highlight that larger dataset sizes make introgression testing methods more susceptible to rate variation.

### False Negatives and Positives Caused by Rate Variation in Introgression Scenarios

In this part, we simulated introgression scenarios (Fig. 7a, Scenario 8 in Table 1). We assumed branch lengths *TS*  *=*  *TI*  *=*  *TO*  *=*  *T* and considered an episodic outflow event from *P1* to *P3* at 0.95*T*, with an introgression proportion of 0.05. This analysis focuses on the role of phylogenetic depth (*T*) and population size (2*N*) on introgression testing. We considered 2 key metrics: the false-negative rate, referring to instances where the true introgression event (*P1*↔*P3*) was not detected, and the false-positive rate, referring to cases where an incorrect introgression event was inferred. Figure 7 and supplementary fig. S7, Supplementary Material online summarize the corresponding results.

When rate variation was absent (i.e. $\lambda_{P1}=1$), *D*-values were negative as expected. The *D*-statistic successfully detected introgression between *P1* and *P3* under many parameter combinations (Fig. 7b and c). HyDe, however, confused the direction of gene flow, misidentifying *P1* as the hybrid lineage, an issue that had already been noted in previous studies (Pang and Zhang 2024). The degree to which *D*-values deviated from 0 depended on both phylogenetic age and population size. For phylogenetic age *T*, the relationship between *D*-values and *T* showed a U-shaped trend (Fig. 7b). Especially in cases with a small population size of 2*N* = 10^4^, the mean *D*-values sharply decreased from −0.0078 to −0.19 as *T* increased from 10^4^ to 5 × 10^4^ and then gradually increased to −0.03 at *T* = 10^6^. The power of tests rose from 5% at *T* = 10^4^ to 100% for *T* ≥ 5 × 10^4^ (Fig. 7c). Regarding population size, both the absolute *D*-values and the test power decreased as 2*N* increased, indicating that true introgression signals are more readily detected in groups with small ancestral population sizes (Fig. 7b and c).

When *P1*’s mutation rate accelerated (i.e. $\lambda_{P1}>1$), *D*-values gradually shifted from negative to 0 and even became positive as $\lambda_{P1}$ increased (Fig. 7b). This trend resulted in a decreased test power, and in some cases, the detection of spurious introgression events (Fig. 7c). This negative effect of rate variation was particularly pronounced in cases with older phylogenies and small population sizes. For example, when *T* = 10^6^ and 2*N* = 10^4^, a 20% increase in the evolutionary rate of *P1* ($\lambda_{P1}=1.2$) led to a failure to detect introgression signals (10% power), while a 50% increase ($\lambda_{P1}=1.5$) resulted in incorrect inference of *P2*↔*P3* gene flow using the *D*-statistic (100% FP rate) (Fig. 7c). In contrast, when the evolutionary rate of *P1* was slower (i.e. $\lambda_{P1}<1$), rate variation had a beneficial effect, enhancing the *ABBA–BABA* asymmetry and improving the power to detect *P1*↔*P3* introgression (supplementary fig. S7, Supplementary Material online).

## Discussion

The widespread use of summary tests for introgression has spurred numerous evaluation studies on their performance, including sensitivity analyses for false negatives (Zheng and Janke 2018; Kong and Kubatko 2021; Bjorner et al. 2024) and robustness assessments against false positives (Tricou et al. 2022; Frankel and Ané 2023; Koppetsch et al. 2024; Pang and Zhang 2024). Recently, attention has been drawn to the commonly assumed molecular clock model in these methods, with simulation studies revealing that rate variation can introduce false-positive signals in deeply divergent groups (Frankel and Ané 2023; Koppetsch et al. 2024). Here, we shift the focus to shallow phylogenies, where rate variation remains widespread and appreciable (Fig. 1) and where introgression testing is most commonly applied. We systematically evaluate the sensitivity of site pattern-based methods—specifically, the *D*-statistic and HyDe—to rate variation under various phylogenetic and demographic conditions.

Our theoretical analysis confirms that rate variation between sister lineages can create asymmetric *ABBA* and *BABA* counts, even in the absence of introgression (Fig. 2a). Specifically, the slower-evolving sister lineage has a higher probability of sharing alleles with *P3*. This may appear counterintuitive and contradicts the conclusions of Frankel and Ané (2023; Figure 8), who suggest that the faster-evolving sister lineage and *P3* are more likely to undergo homoplasies and share alleles. The discrepancy arises because Frankel and Ané (2023) ignored mutations along the outgroup *O*, which necessarily occur more frequently than those along *P3*. As a consequence, rate variation-induced *ABBA–BABA* imbalance is misinterpreted as a signal of gene flow, with the slower-evolving sister lineage mistakenly identified as a participant in an introgression event by the *D*-statistic and as a hybrid lineage by HyDe. Furthermore, in cases with introgression, rate variation can alter the magnitude or even the sign of the *D*-value, potentially leading to both false negatives and false positives (Fig. 7).

**Fig. 6.**
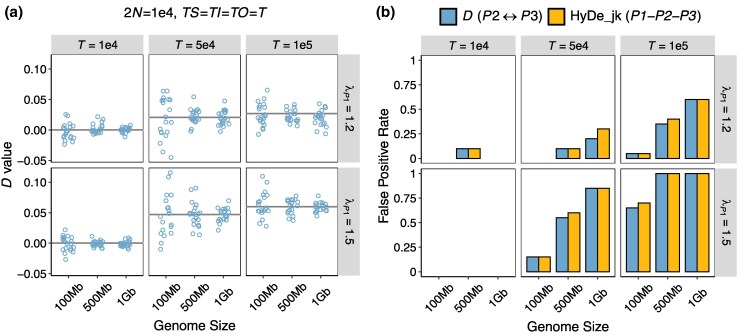
Effects of dataset size on summary tests for introgression. The simulated demographic scenario corresponds to Fig. 3a. Branch lengths are fixed at *TS* = *TI* = *TO* = *T*, and $2N=10^{4}$. The values on the strips at the top and right of each plot indicate the phylogenetic depth (*T*) and the extent of rate variation ($\lambda_{P1}$), respectively. Genome size is labeled on the *x*-axis. a) *D*-values: Colored points represent *D* estimates, with solid lines indicating the theoretical expected *D*. b) False-positive rate of the *D*-statistic and HyDe_jk. See Fig. 3 for an explanation of the symbol legends.

The interaction between phylogeny age and population size modulates the risk of false positives caused by rate variation. Our results support the intuition that introgression tests performed for older groups are more vulnerable to rate variation (Fig. 3 and supplementary fig. S5, Supplementary Material online). Nevertheless, phylogenetic age is not the sole determinant—population size also exerts a substantial influence. Specifically, small population sizes (in ancestral lineages) can render even shallow phylogenies sensitive to rate variation, producing high *D*-values and false-positive signals. For example, in young groups with a phylogeny age of 3 × 10^5^ generations (corresponding to *T* = 10^5^ in Fig. 3 and supplementary fig. S5, Supplementary Material online) and a small population size of 2*N* = 10^4^, even a weak 17% rate variation between sister lineages (i.e. λ=1.2) results in FP rates of 35% for 500 Mb datasets, rising to 60% for 1 Gb datasets (Fig. 6b). A moderate 33% rate difference (i.e. *λ* = 1.5) produces *D*-values exceeding 0.05, comparable to the 0.047 *D*-value reported in the Neanderthal-modern human introgression study by Green et al. (2010). Under this condition, FP rates surpass 50% in a 100 Mb dataset (Fig. 3 and supplementary fig. S5, Supplementary Material online) and reach 100% when the dataset size increases to 500 Mb (Fig. 6). Interestingly, a previous work by Zheng and Janke (2018) also revealed the pivotal role of population size in the power of the *D*-statistic when introgression is present, showing a significant negative correlation between the two. In conclusion, both false and true introgression signals are stronger in groups with smaller population sizes. Larger population sizes, in contrast, produce higher ILS-driven *ABBA* and *BABA* counts by increasing the frequencies of discordant gene trees and elongating their internal branches, obscuring asymmetric signals caused by either rate variation or actual introgression.

**Fig. 7.**
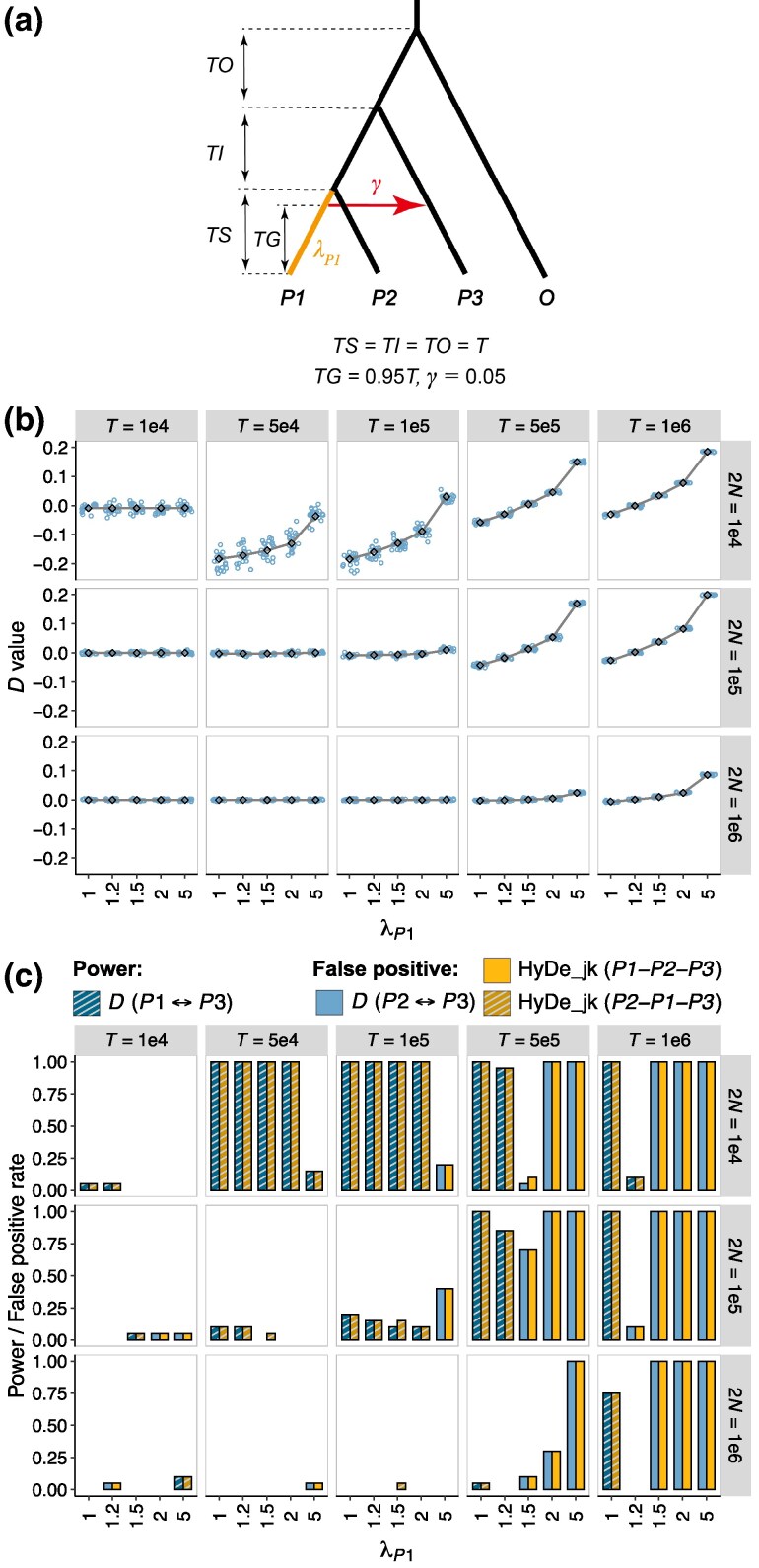
Effect of rate variation on summary tests in the presence of introgression. a) Simulated demographic histories. Branch lengths are measured in generations, with *TS* = *TI* = *TO* = *T*. An outflow introgression (*P1* → *P3*) occurs at 0.95*T* with a proportion *γ* of 0.05. $\lambda_{P1}$ represents the relative rate of lineage *P1*. b and c). Results of introgression tests. The values on the strips at the top and right of each plot indicate the phylogenetic depth (*T*) and population size (2*N*), respectively. $\lambda_{P1}$ is labeled on the x-axis. b) *D*-values: Colored points represent *D*-value estimates, with solid lines indicating the theoretically expected *D*-values. c) Power or false-positive rate of the *D*-statistic and HyDe_jk. The *y*-axis shows power when the introgression between *P1* and *P3* is correctly detected, and the false-positive rate when a nonexistent introgression is inferred incorrectly. See Fig. 3 for an explanation of the symbol legends.

Outgroup distance has a large impact on the incidence of false positives induced by rate heterogeneity. In empirical studies, the outgroup is typically selected to ensure sufficient genetic divergence from the ingroups to mitigate the risk of introgression between them (Green et al. 2010). Zheng and Janke (2018) justified this practice, suggesting that the power of the *D*-statistic seems unaffected by outgroup distance. However, their simulations did not account for rate variation among lineages. Our study shows that a more distant outgroup can largely increase the likelihood of false positives arising from rate variation (Figs. 2d, 4e and f). For instance, in the scenarios discussed earlier (*TS* = *TI* = *TO* = 10^5^ generations and *λ* = 1.5), increasing the outgroup distance *TO* to 10^6^ generations leads to a striking increase in the *D*-value to around 0.15, alongside an FP rate of 100% based on a 100 Mb dataset (Fig. 4e and f). This occurs because a larger branch difference between *P3* and the outgroup *O* amplifies *ABBA–BABA* asymmetry resulting from homoplasies. Due to the same underlying mechanism, a reduced evolutionary rate of *P3* or an accelerated rate in the outgroup *O* can also produce a similar effect of exacerbating FP rates (Fig. 5). Our findings highlight the challenges in selecting an appropriate outgroup, necessitating careful consideration of the trade-offs between distant and closely related outgroups. We lean toward recommending the selection of a closely related outgroup, as this can yield higher-quality data due to fewer mapping and alignment errors, while also reducing false positives caused by rate variation. On the other hand, researchers should also be mindful of the potential for introgression involving the outgroup when interpreting significant results.

Our study did not evaluate methods that rely on gene-tree branch lengths or those that depend exclusively on gene-tree topologies. Intuitively, the former is more susceptible to deviations from the clock assumption, while the latter tends to be more robust (Frankel and Ané 2023; Koppetsch et al. 2024). This expectation stems from the fact that rate variation does not alter the coalescent trajectories of alleles that shape the topology but rather affects mutation accumulation along those trajectories, which determines branch lengths. For methods relying on branch length information, rate variation causes deviations in branch lengths from those expected under ILS, resulting in false signals of introgression as the method seeks to reconcile these inconsistencies. For example, Frankel and Ané (2023) found that *D_3_* is particularly vulnerable to rate variation between 2 sister lineages. We anticipate that full-likelihood tests would also exhibit high sensitivity to rate heterogeneity, which warrants further investigation to confirm. As for topology-based methods, their performance depends heavily on the accuracy of gene-tree topology inference. These methods may perform well for deep phylogenies but are less suitable for shallow phylogenies where the sparse phylogenetic signal per locus impedes accurate topological reconstruction. For example, loci exhibiting only a single parsimony-informative site, if arising from homoplasy, inevitably produce false topologies due to long-branch attraction.

### Implications for Practice

In empirical systems, researchers may wonder under what conditions introgression testing is most susceptible to rate variation and how to differentiate it from genuine introgression signals. For a given group of closely related taxa, we recommend first quantifying the extent of rate variation using the relative rate test, as applied to the 6 genera in the Introduction section of this study (Fig. 1). When species pairs differ in evolutionary rates by over 20%, caution is especially needed—particularly if their ancestral population sizes are small—since these factors together significantly elevate false-positive risks. Additionally, we advise conducting simulations tailored to the group’s specific characteristics, including the extent of rate variation, divergence times, population size, and genome size. Such simulations can offer a more nuanced view of rate variation's impact on the group in question.

Several strategies may help researchers distinguish between patterns driven by rate variation and those caused by genuine introgression. A key feature of rate variation-driven signals is that the sister lineage with the shorter branch length tends to have a higher probability of sharing alleles with *P3*. Therefore, if a test detects introgression between the faster-evolving sister lineage and *P3*, this strongly suggests that the signal reflects genuine introgression. Furthermore, Bayesian model comparison offers a rigorous framework for evaluating the evidence for gene flow in light of violating the clock assumption. Species tree methods, which are well-established and flexible, can accommodate variable assumptions about rate variation across lineages. A practical approach involves comparing 2 models under a full-likelihood framework: (i) a species tree model that accounts for rate heterogeneity (e.g. a relaxed clock model) and (ii) an introgression model that explicitly incorporates gene flow. If the introgression model demonstrates a significantly higher marginal likelihood than the rate heterogeneity model, this outcome provides statistical support for the presence of gene flow. However, this strategy is computationally demanding and may be infeasible for large genome-scale datasets.

Koppetsch et al. (2024) recently developed a user-friendly “*ABBA*-site clustering” test to distinguish between spurious and genuine introgression signals, based on the distribution of *D*-informative sites across the genome. The test capitalizes on the fact that introgression typically leaves behind haplotypes with clusters of linked “*ABBA*” sites that reflect introgression history, while sites arising from homoplasies are expected to be randomly distributed. By identifying *ABBA*-clustering patterns along chromosomes, the test can effectively detect introgression in deep phylogenies. However, mutation hotspots or mapping biases can also generate clustered “*ABBA*” sites. To address this, Koppetsch et al. (2024) developed a more “robust” version of the test, but it exhibited high false-negative rates even under strong introgression. Additionally, its performance in shallow phylogenies remains uncertain, where mutations are rarer and introgression-driven *ABBA* clusters may be difficult to detect.

Given these limitations, there is a pressing need for more refined and powerful methods to differentiate signals arising from rate variation and genuine introgression. Until such methods are developed, empirical researchers should adopt a conservative stance, considering rate variation as one of the default possibilities when interpreting significant results from introgression testing methods, unless compelling evidence suggests otherwise.

## Supplementary Material

msaf216_Supplementary_Data

## Data Availability

Simulated datasets are available in Zenodo at https://doi.org/10.5281/zenodo.16886629.
