# Supplementary material for: Detecting Introgression in Shallow Phylogenies: How Minor Molecular Clock Deviations Lead to Major Inference Errors
Source: Mol Biol Evol. 2025 Sep 10;42(10):msaf216. doi: 10.1093/molbev/msaf216 (PMC12485364; doi:10.1093/molbev/msaf216)
Supplement: msaf216_Supplementary_Data [file msaf216_supplementary_data.pdf]

Supplementary Note 1

We employed the relative rate test (Graur and Li 2000) to quantify the extent of rate variation between species pairs. Specifically, we used an outgroup, *O*, as reference. For each lineage pair within the ingroups, *A* and *B*, we calculated the branch lengths of *AI* and *BI* for each locus, based on genetic distances of the sequence pairs K_{AB}, K_{AO}, and K_{BO}, as described in Figure S1. The genetic distances were calculated using the *dist.dna* function from the **ape** R package, with the JC69 model and the option pairwise.deletion = TRUE. Then we computed the mean value of K_{AI} and K_{BI} across all loci, with their relative difference representing the rate difference of the species pair *A* and *B*.

It should be noted that the branch lengths of *AI* and *BI* for each locus include a segment corresponding to the common ancestor of species *A* and *B*, where the evolutionary rate is shared. Therefore, the relative rate test method tends to underestimate rate difference.

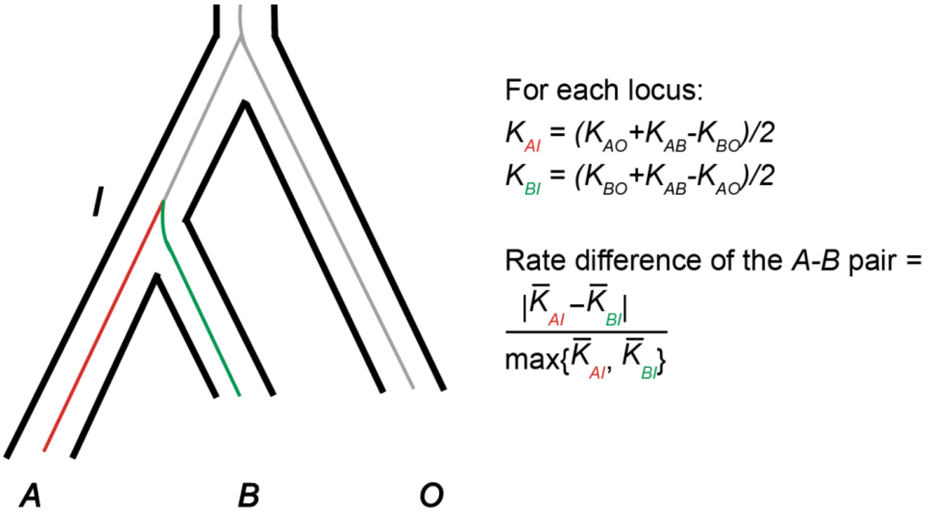

Figure S1. Calculation of rate difference between species pairs (Graur and Li 2000). The value $\overline{K}$ represents the mean over all loci.

Table S1. Details of the Six Genera Datasets

Genera	Number of species	Number of Loci	Literatures
Adansonia	8	372	(Karimi et al. 2020)
Ficus	26	1477	(Gardner et al. 2023)
Habronattus	34	1877	(Leduc-Robert and Maddison 2018)
Jaltomata	14	1000	(Wu et al. 2018)
Malus	13	620	(Liu et al. 2022)
Tamias	6	1060	(Sarver et al. 2021)

Supplementary Note 2

For the MSci model *N* shown in Figure S2a, the site-pattern frequencies can be obtained by integrating over all possible gene-tree histories:

$$P(site|N,\lambda) = \int_{\{G,t\}} f\big(site\big|G,t,\lambda\big) \times f\big((G,t)|N\{\theta,\tau_1,\tau_2,\tau_3,\tau_g,\gamma\}\big) \qquad (S1)$$

where the *site* corresponds to one of the *BBAA*, *ABBA*, or *BABA* patterns. The terms $f(\{G,t\}|N\}$ and $f(site|G,t,\lambda)$ represent the coalescent process and the sequence evolution process, respectively, where $t = \{t_1, t_2, t_3\}$ denotes the coalescent times of sequences within the gene tree *G* (see Figs. S2b-c).

We begin with the coalescent process, with one sample per lineage (Figs. S2b-c). Since there is only one descendant at the hybrid node in the model *N*, the coalescent process can be represented as a mixture over two parental trees *S* and *I* (Fig. S2a), each corresponding to one of the hybrid edges, with probabilities γ and $1-\gamma$, respectively (Meng and Kubatko 2009; Zhu and Degnan 2017):

$$\begin{aligned} &f((G,t)|N\{\theta,\tau_1,\tau_2,\tau_3,\tau_g\} \\ &= (1-\gamma)f((G,t)|S\{\theta,\tau_1,\tau_2,\tau_3\}) + \gamma f((G,t)|I\{\theta,\tau_g,\tau_1,\tau_3,\}) \end{aligned} \qquad (S2)$$

For the parental tree *S*: (((*P1*, *P2*):τ_1, *P3*):τ_2, *O*):τ_3, gene trees can be classified into 5 categories A_S-E_S based on the number of coalescent events occurring within the time interval τ_1—τ_2, τ_2—τ_3, and >τ_3 (Fig. S2b). Similarly, for the parental tree ***I***: (((*P1*, *P3*):τ_g, *P2*):τ_1, *O*):τ_3, gene tree histories are also classified into 5 categories A_I-E_I based on coalescent events within the time interval τ_g—τ_1, τ_1—τ_3, and >τ_3 (Fig. S2c). The probability densities for these gene trees, characterized by coalescent times t_1, t_2, and t_3, follow the framework of Rannala and Yang (2003) and are detailed in Table S2.

Next, we consider the process of substitutions along gene trees (Figs. S2b-c). When the relative rate parameter $\lambda = 1$ (i.e., in the absence of rate variation), the external branch lengths of unrooted gene trees—b_{p_1}, b_{p_2}, b_{p_3}, and b_o—as well as the internal branch length b_i can be directly determined from the coalescent times t_i. However, when $\lambda \neq 1$, the branch segments of gene trees that pass through lineage *P1* are scaled by the factor λ. Specifically, the branch lengths are adjusted from their values under $\lambda = 1$ as follows:

For gene trees A_S-E_S:

$$b_{p_1} \to b_{p_1} + (\lambda - 1)\tau_1 \qquad (S3)$$

For gene trees A_I-B_I:

$$\begin{cases} b_{p_1} \to b_{p_1} + (\lambda - 1)t_1 \\ b_{p_3} \to b_{p_3} + (\lambda - 1)(t_1 - \tau_g) \\ b_i \to b_i + (\lambda - 1)(\tau_1 - t_1) \end{cases} \qquad (S4)$$

For gene trees C_I-D_I:

$$\begin{cases} b_{p_1} \to b_{p_1} + (\lambda - 1)\tau_1 \\ b_{p_3} \to b_{p_3} + (\lambda - 1)(\tau_1 - \tau_g) \end{cases} \qquad (S5)$$

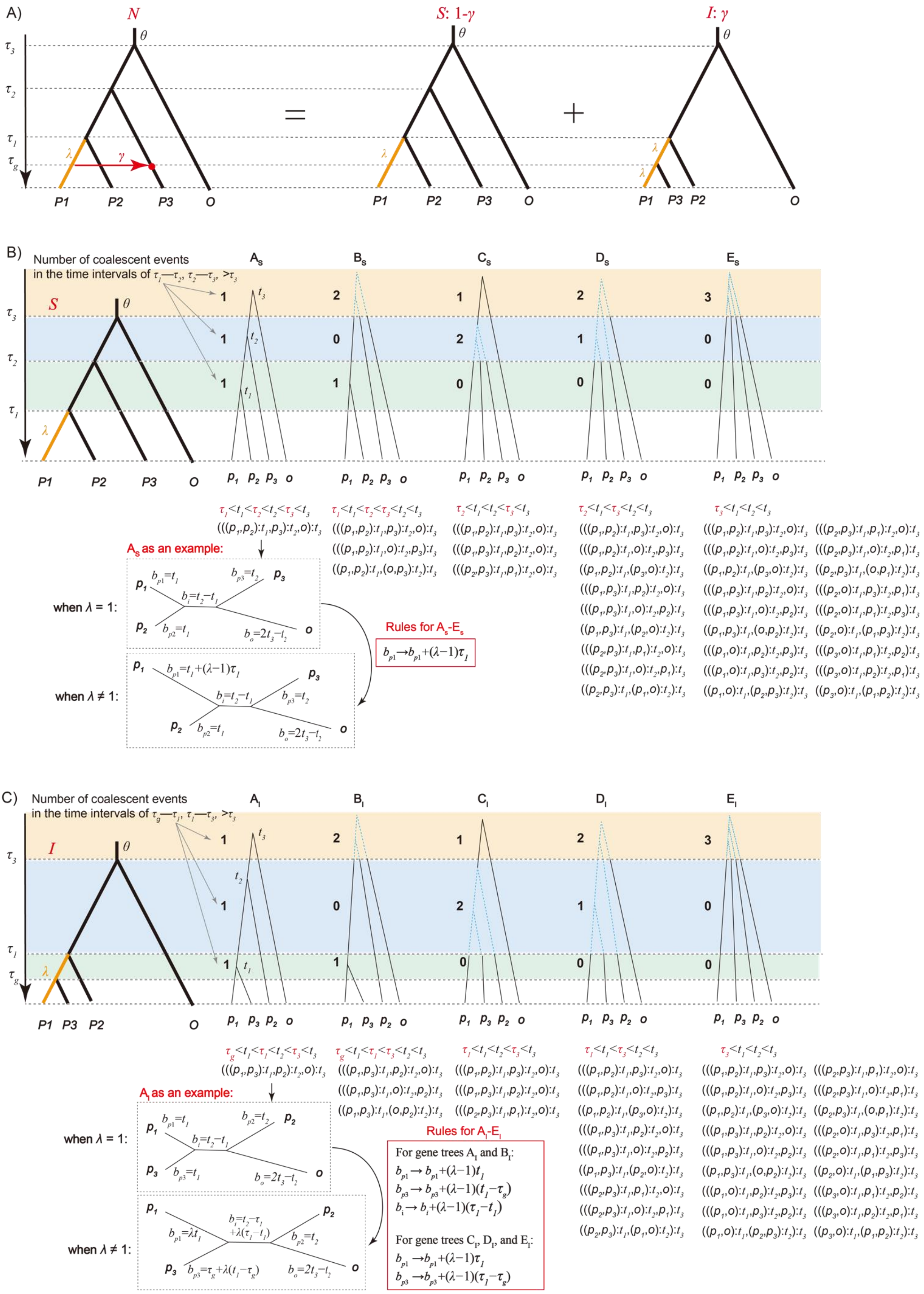
A)
N
S: 1-γ
I: γ
θ
τ_3
τ_2
τ_1
τ_g
λ
γ
=
+
P1 P2 P3 O
P1 P3 P2 O
B)
Number of coalescent events
in the time intervals of τ_1—τ_2, τ_2—τ_3, >τ_3
S
A_S B_S C_S D_S E_S
p_1 p_2 p_3 o
$\tau_1<t_1<\tau_2<t_2<\tau_3<t_3$
$(((p_1,p_2):t_1,p_3):t_2,o):t_3$
$\tau_1<t_1<\tau_2<\tau_3<t_2<t_3$
$(((p_1,p_2):t_1,p_3):t_2,o):t_3$
$(((p_1,p_2):t_1,o):t_2,p_3):t_3$
$((p_1,p_2):t_1,(o,p_3):t_2):t_3$
$\tau_2<t_1<t_2<\tau_3<t_3$
$(((p_1,p_2):t_1,p_3):t_2,o):t_3$
$(((p_1,p_3):t_1,p_2):t_2,o):t_3$
$(((p_2,p_3):t_1,p_1):t_2,o):t_3$
$\tau_2<t_1<\tau_3<t_2<t_3$
$\tau_3<t_1<t_2<t_3$
A_S as an example:
when λ = 1:
when λ ≠ 1:
$b_{p1}=t_1$
$b_{p2}=t_1$
$b_i=t_2-t_1$
$b_{p3}=t_2$
$b_o=2t_3-t_2$
$b_{p1}=t_1+(\lambda-1)\tau_1$
Rules for A_S-E_S
$b_{p1}\rightarrow b_{p1}+(\lambda-1)\tau_1$
C)
Number of coalescent events
in the time intervals of τ_g—τ_1, τ_1—τ_3, >τ_3
I
A_I B_I C_I D_I E_I
p_1 p_3 p_2 o
$\tau_g<t_1<\tau_1<t_2<\tau_3<t_3$
$(((p_1,p_3):t_1,p_2):t_2,o):t_3$
$\tau_g<t_1<\tau_1<\tau_3<t_2<t_3$
$(((p_1,p_3):t_1,p_2):t_2,o):t_3$
$(((p_1,p_3):t_1,o):t_2,p_2):t_3$
$((p_1,p_3):t_1,(o,p_2):t_2):t_3$
$\tau_1<t_1<t_2<\tau_3<t_3$
$(((p_1,p_2):t_1,p_3):t_2,o):t_3$
$(((p_1,p_3):t_1,p_2):t_2,o):t_3$
$(((p_2,p_3):t_1,p_1):t_2,o):t_3$
$\tau_1<t_1<\tau_3<t_2<t_3$
$\tau_3<t_1<t_2<t_3$
A_I as an example:
when λ = 1:
when λ ≠ 1:
$b_{p1}=t_1$
$b_{p3}=t_1$
$b_i=t_2-t_1$
$b_{p2}=t_2$
$b_o=2t_3-t_2$
$b_{p1}=\lambda t_1$
$b_i=t_2-\tau_1+\lambda(\tau_1-t_1)$
$b_{p3}=\tau_g+\lambda(t_1-\tau_g)$
Rules for A_I-E_I
For gene trees A_I and B_I:
$b_{p1}\rightarrow b_{p1}+(\lambda-1)t_1$
$b_{p3}\rightarrow b_{p3}+(\lambda-1)(t_1-\tau_g)$
$b_i\rightarrow b_i+(\lambda-1)(\tau_1-t_1)$
For gene trees C_I, D_I, and E_I:
$b_{p1}\rightarrow b_{p1}+(\lambda-1)\tau_1$
$b_{p3}\rightarrow b_{p3}+(\lambda-1)(\tau_1-\tau_g)$

Figure S2. A) Decomposition of network into parental species trees. The hybrid node is depicted as the circular point located on the right side of the arrow. The introgression scenario *N* depicted on the left contains 2 parental species trees *S* and *I* at the middle and right. The probabilities assigned to the 2 parental species trees are γ and $1-\gamma$. B) Possible gene-tree histories under the parental tree *S*. Gene trees are categorized into 5 classes (A_S–E_S) based on the number of coalescent events within the time intervals τ_1–τ_2, τ_2–τ_3, and $>\tau_3$. Note that categories B_S–E_S each include multiple coalescent histories; the genealogical trees on the right illustrate one representative coalescent history for each category, while alternative topologies are shown below in Newick format. A schematic in the lower right corner illustrates how gene tree branch lengths are adjusted when $\lambda \neq 1$ (red box), using the gene tree in A_S category as an example (dotted box). C) Possible gene-tree histories under the parental tree *I*.

Table S2. Probability density of gene trees for the parental trees *S* and *I*

Class	Number of the gene trees	Probability density for each gene tree under the MSci model *N*
**A_S**	1	$(1-\gamma)(\frac{2}{\theta})^3 exp\{-\frac{2}{\theta}(t_1-\tau_1)-\frac{2}{\theta}(t_2-\tau_2)-\frac{2}{\theta}(t_3-\tau_3)\}$
**B_S**	3	$(1-\gamma)(\frac{2}{\theta})^3 exp\{-\frac{2}{\theta}(t_1-\tau_1)-\frac{2}{\theta}(t_3-\tau_2)-\frac{6}{\theta}(t_2-\tau_3)-\frac{2}{\theta}(t_3-t_2)\}$
**C_S**	3	$(1-\gamma)(\frac{2}{\theta})^3 exp\{-\frac{2}{\theta}(\tau_2-\tau_1)-\frac{6}{\theta}(t_1-\tau_2)-\frac{2}{\theta}(t_2-t_1)-\frac{2}{\theta}(t_3-\tau_3)\}$
**D_S**	9	$(1-\gamma)(\frac{2}{\theta})^3 exp\{-\frac{2}{\theta}(\tau_2-\tau_1)-\frac{6}{\theta}(t_1-\tau_2)-\frac{2}{\theta}(\tau_2-t_1)-\frac{2}{\theta}(t_3-\tau_3)\}$
**E_S**	18	$(1-\gamma)(\frac{2}{\theta})^3 exp\{-\frac{2}{\theta}(\tau_2-\tau_1)-\frac{6}{\theta}(\tau_3-\tau_2)-\frac{12}{\theta}(t_1-\tau_3)-\frac{6}{\theta}(t_2-t_1)-\frac{2}{\theta}(t_3-t_2)\}$
**A_I**	1	$\gamma(\frac{2}{\theta})^3 exp\{-\frac{2}{\theta}(t_1-\tau_g)-\frac{2}{\theta}(t_2-\tau_1)-\frac{2}{\theta}(t_3-\tau_3)\}$
**B_I**	3	$\gamma(\frac{2}{\theta})^3 exp\{-\frac{2}{\theta}(t_1-\tau_g)-\frac{2}{\theta}(t_3-\tau_1)-\frac{6}{\theta}(t_2-\tau_3)-\frac{2}{\theta}(t_3-t_2)\}$
**C_I**	3	$\gamma(\frac{2}{\theta})^3 exp\{-\frac{2}{\theta}(\tau_1-\tau_g)-\frac{6}{\theta}(t_1-\tau_1)-\frac{2}{\theta}(t_2-t_1)-\frac{2}{\theta}(t_3-\tau_3)\}$
**D_I**	9	$\gamma(\frac{2}{\theta})^3 exp\{-\frac{2}{\theta}(\tau_1-\tau_g)-\frac{6}{\theta}(t_1-\tau_1)-\frac{2}{\theta}(\tau_2-t_1)-\frac{2}{\theta}(t_3-\tau_3)\}$
**E_I**	18	$\gamma(\frac{2}{\theta})^3 exp\{-\frac{2}{\theta}(\tau_1-\tau_g)-\frac{6}{\theta}(\tau_3-\tau_1)-\frac{12}{\theta}(t_1-\tau_3)-\frac{6}{\theta}(t_2-t_1)-\frac{2}{\theta}(t_3-t_2)\}$

The probabilities of parsimony-informative site patterns for a given unrooted gene tree *UG* under JC69 model have been derived by Richards and Kubatko (2022):

$$f(BBAA|UG\{b_{p_1},b_{p_2},b_{p_3},b_o,b_i\}\}=\frac{1}{256}(1+3e^{-(b_{p_1}+b_{p_2})}+3e^{-(b_{p_3}+b_o)}-e^{-(b_{p_1}+b_{p_3}+b_i)}-e^{-(b_{p_1}+b_o+b_i)}-e^{-(b_{p_2}+b_{p_3}+b_i)}-e^{-(b_{p_2}+b_o+b_i)}+9e^{-(b_{p_1}+b_{p_2}+b_{p_3}+b_o)}-2e^{-(b_{p_1}+b_{p_2}+b_{p_3}+b_i)}-2e^{-(b_{p_1}+b_{p_2}+b_o+b_i)}-2e^{-(b_{p_1}+b_{p_3}+b_o+b_i)}-2e^{-(b_{p_2}+b_{p_3}+b_o+b_i)}-4e^{-(b_{p_1}+b_{p_2}+b_{p_3}+b_o+b_i)})$$

$$f(BABA|UG\{b_{p_1}, b_{p_2}, b_{p_3}, b_o, b_i\}\} = \frac{1}{256}(1 - e^{-(b_{p_1}+b_{p_2})} - e^{-(b_{p_3}+b_o)} + 3e^{-(b_{p_1}+b_{p_3}+b_i)} - e^{-(b_{p_1}+b_o+b_i)} - e^{-(b_{p_2}+b_{p_3}+b_i)} + 3e^{-(b_{p_2}+b_o+b_i)} + e^{-(b_{p_1}+b_{p_2}+b_{p_3}+b_o)} - 2e^{-(b_{p_1}+b_{p_2}+b_{p_3}+b_i)} - 2e^{-(b_{p_1}+b_{p_2}+b_o+b_i)} - 2e^{-(b_{p_1}+b_{p_3}+b_o+b_i)} - 2e^{-(b_{p_2}+b_{p_3}+b_o+b_i)} + 4e^{-(b_{p_1}+b_{p_2}+b_{p_3}+b_o+b_i)})$$

$$f(ABBA|UG\{b_{p_1}, b_{p_2}, b_{p_3}, b_o, b_i\}\} = \frac{1}{256}(1 - e^{-(b_{p_1}+b_{p_2})} - e^{-(b_{p_3}+b_o)} - e^{-(b_{p_1}+b_{p_3}+b_i)} + 3e^{-(b_{p_1}+b_o+b_i)} + 3e^{-(b_{p_2}+b_{p_3}+b_i)} - e^{-(b_{p_2}+b_o+b_i)} + e^{-(b_{p_1}+b_{p_2}+b_{p_3}+b_o)} - 2e^{-(b_{p_1}+b_{p_2}+b_{p_3}+b_i)} - 2e^{-(b_{p_1}+b_{p_2}+b_o+b_i)} - 2e^{-(b_{p_1}+b_{p_3}+b_o+b_i)} - 2e^{-(b_{p_2}+b_{p_3}+b_o+b_i)} + 4e^{-(b_{p_1}+b_{p_2}+b_{p_3}+b_o+b_i)}) \quad (S6)$$

By considering all gene tree histories, we substitute their probability densities (Table S2) and the conditional probabilities of site patterns for a given gene tree (Eq. S6) into Equation S1. Integrating over the coalescent times yields the probabilities of the parsimony-informative site patterns. The final expressions are provided in Table S3.

Table S3. The probabilities of parsimony-informative site patterns for the MSci model *N*.

**Exponential terms**	**Coefficients**		
	BBAA	***BABA***	***ABBA***
1	$\frac{3}{64}$	$\frac{3}{64}$	$\frac{3}{64}$
$e^{-\frac{8\tau_g}{3}}$	$-\frac{3k'^{-1}\gamma}{64(1+\lambda a)}$	$\frac{9k'^{-1}\gamma}{64(1+\lambda a)}$	$-\frac{3k'^{-1}\gamma}{64(1+\lambda a)}$
$e^{-\frac{8\tau_1}{3}}$	$\frac{3k(3-\gamma k')}{64(1+a)}$	$-\frac{3k(1+\gamma k')}{64(1+a)}$	$\frac{3k(3\gamma k'-1)}{64(1+a)}$
$e^{-\frac{8\tau_2}{3}}$	$-\frac{3(1+k)(1-\gamma)}{64(1+a)}$	$\frac{3(3k-1)(1-\gamma)}{64(1+a)}$	$\frac{3(3-k)(1-\gamma)}{64(1+a)}$
$e^{-\frac{8\tau_3}{3}}$	$\frac{3(2-k)+9\gamma(kk'-1)}{64(1+a)}$	$\frac{3(-kk'\gamma+\gamma-k+2)}{64(1+a)}$	$\frac{3(-kk'\gamma+\gamma+3k-2)}{64(1+a)}$
$e^{-\frac{4}{3}(\tau_g+2\tau_1)}$	$-\frac{3k\gamma}{16(1+a)(2+\lambda a)}$	$-\frac{3k\gamma}{16(1+a)(2+\lambda a)}$	$-\frac{3k\gamma}{16(1+a)(2+\lambda a)}$
$e^{-\frac{4}{3}(\tau_g+2\tau_3)}$	$-\frac{3k\gamma}{16(1+a)(2+\lambda a)}$	$-\frac{3k\gamma}{16(1+a)(2+\lambda a)}$	$-\frac{3k\gamma}{16(1+a)(2+\lambda a)}$
$e^{-\frac{4}{3}(\tau_1+2\tau_2)}$	$-\frac{3k(1-\gamma)}{16(1+a)(2+a)}$	$-\frac{3k(1-\gamma)}{16(1+a)(2+a)}$	$-\frac{3k(1-\gamma)}{16(1+a)(2+a)}$
$e^{-\frac{4}{3}(\tau_1+2\tau_3)}$	$-\frac{3k(1+k'\gamma)}{16(1+a)(2+a)}$	$-\frac{3k(1+k'\gamma)}{16(1+a)(2+a)}$	$-\frac{3k(1+k'\gamma)}{16(1+a)(2+a)}$
$e^{-\frac{4}{3}(\tau_2+2\tau_3)}$	$-\frac{3(1+k)(1-\gamma)}{16(1+a)(2+a)}$	$-\frac{3(1+k)(1-\gamma)}{16(1+a)(2+a)}$	$-\frac{3(1+k)(1-\gamma)}{16(1+a)(2+a)}$

$e^{-\frac{4}{3}(2\tau_g+2\tau_3)}$	$\frac{3k'^{-1}\gamma}{64(1+a)(1+\lambda a)}$	$\frac{27k'^{-1}\gamma}{64(1+a)(1+\lambda a)}$	$\frac{3k'^{-1}\gamma}{64(1+a)(1+\lambda a)}$
$e^{-\frac{4}{3}(2\tau_1+2\tau_3)}$	$\frac{27k(1-\gamma)}{64(1+a)^2}$	$\frac{3k(1-\gamma)}{64(1+a)^2}$	$\frac{3k(1-\gamma)}{64(1+a)^2}$
$e^{-\frac{4}{3}(\tau_g+\tau_1+2\tau_3)}$	$\frac{3k\gamma}{4(1+a)(2+a)(2+\lambda a)}$	$-\frac{3k\gamma}{4(1+a)(2+a)(2+\lambda a)}$	$\frac{3k\gamma}{4(1+a)(2+a)(2+\lambda a)}$
$e^{-\frac{4}{3}(\tau_1+\tau_2+2\tau_3)}$	$-\frac{3k(1-\gamma)}{4(1+a)(2+a)^2}$	$\frac{3k(1-\gamma)}{4(1+a)(2+a)^2}$	$\frac{3k(1-\gamma)}{4(1+a)(2+a)^2}$
$e^{-\frac{2(\tau_1-\tau_g)}{\theta}}e^{-\frac{8\tau_1}{3}}$	$-\frac{3(\lambda-1)ak^2k'\gamma}{64(1+a)(1+\lambda a)}$	$\frac{9(\lambda-1)ak^2k'\gamma}{64(1+a)(1+\lambda a)}$	$-\frac{3(\lambda-1)ak^2k'\gamma}{64(1+a)(1+\lambda a)}$
$e^{-\frac{2(\tau_1-\tau_g)}{\theta}}e^{-\frac{12\tau_1}{3}}$	$-\frac{3(\lambda-1)ak^2k'\gamma}{16(1+a)(2+a)(2+\lambda a)}$	$-\frac{3(\lambda-1)ak^2k'\gamma}{16(1+a)(2+a)(2+\lambda a)}$	$-\frac{3(\lambda-1)ak^2k'\gamma}{16(1+a)(2+a)(2+\lambda a)}$
$e^{-\frac{2(\tau_1-\tau_g)}{\theta}}e^{-\frac{4(\tau_1+2\tau_3)}{3}}$	$-\frac{3(\lambda-1)ak^2k'\gamma}{16(1+a)(2+a)(2+\lambda a)}$	$-\frac{3(\lambda-1)ak^2k'\gamma}{16(1+a)(2+a)(2+\lambda a)}$	$-\frac{3(\lambda-1)ak^2k'\gamma}{16(1+a)(2+a)(2+\lambda a)}$
$e^{-\frac{2(\tau_2-\tau_1)}{\theta}-\frac{4}{3}(2\tau_2+2\tau_3)}$	$\frac{3ak(4+a)^2(1-\gamma)}{32(1+a)^2(2+a)^2(3+a)}$	$\frac{3ak(32+40a+10a^2)(1-\gamma)}{64(1+a)^2(2+a)^2(3+a)}$	$\frac{3ak(32+40a+10a^2)(1-\gamma)}{64(1+a)^2(2+a)^2(3+a)}$
$e^{-\frac{2(\tau_1-\tau_g)}{\theta}}e^{-\frac{4}{3}(2\tau_1+2\tau_3)}$	$3ak^2k'\gamma\big(-2(2+a)+\lambda^2a(30+11a)+\lambda(36+12a-a^2)\big)/64(1+a)(2+a)(3+a)(1+\lambda a)(2+\lambda a)$	$3ak^2k'\gamma\big(-2(26+9a)+\lambda^2a(30+11a)+\lambda(84+4a-9a^2)\big)/64(1+a)(2+a)(3+a)(1+\lambda a)(2+\lambda a)$	$3ak^2k'\gamma\big(-2(2+a)+\lambda^2a(30+11a)+\lambda(36+12a-a^2)\big)/64(1+a)(2+a)(3+a)(1+\lambda a)(2+\lambda a)$

Note: $k = e^{-(4/3)(\lambda-1)\tau_1}, k' = e^{-(4/3)(1-\lambda)\tau_g}$, $\mathrm{a} = 4\theta/3$. The full expression is a sum of products of exponential terms and their corresponding coefficients.

Supplementary Note 3

When $\gamma = 0$, there is:

$$
\begin{aligned}
P(BBAA) = \frac{3}{64}\Big(1 &+ \frac{3k}{1+a} e^{-\frac{8\tau_1}{3}} - \frac{1+k}{(1+a)} e^{-\frac{8\tau_2}{3}} + \frac{2-k}{(1+a)} e^{-\frac{8\tau_3}{3}} - \frac{4k}{(1+a)(2+a)} e^{-\frac{4}{3}(\tau_1+2\tau_2)} \\
&- \frac{4k}{(1+a)(2+a)} e^{-\frac{4}{3}(\tau_1+2\tau_3)} - \frac{4(1+k)}{(1+a)(2+a)} e^{-\frac{4}{3}(\tau_2+2\tau_3)} + \frac{9k}{(1+a)^2} e^{-\frac{4}{3}(2\tau_1+2\tau_3)} \\
&- \frac{16k}{(1+a)(2+a)^2} e^{-\frac{4}{3}(\tau_1+\tau_2+2\tau_3)} + \frac{2a(4+a)^2 k}{(1+a)^2(2+a)^2(3+a)} e^{-\frac{2(\tau_2-\tau_1)}{\theta}-\frac{4}{3}(2\tau_2+2\tau_3)}\Big)
\end{aligned}
$$

$$
\begin{aligned}
P(ABBA) = \frac{3}{64}\Big(1 &- \frac{k}{(1+a)} e^{-\frac{8\tau_1}{3}} + \frac{3-k}{(1+a)} e^{-\frac{8\tau_2}{3}} + \frac{3k-2}{(1+a)} e^{-\frac{8\tau_3}{3}} - \frac{4k}{(1+a)(2+a)} e^{-\frac{4}{3}(\tau_1+2\tau_2)} \\
&- \frac{4k}{(1+a)(2+a)} e^{-\frac{4}{3}(\tau_1+2\tau_3)} - \frac{4(1+k)}{(1+a)(2+a)} e^{-\frac{4}{3}(\tau_2+2\tau_3)} + \frac{k}{(1+a)^2} e^{-\frac{4}{3}(2\tau_1+2\tau_3)} \\
&+ \frac{16k}{(1+a)(2+a)^2} e^{-\frac{4}{3}(\tau_1+\tau_2+2\tau_3)} + \frac{a(32+40a+10a^2)k}{(1+a)^2(2+a)^2(3+a)} e^{-\frac{2(\tau_2-\tau_1)}{\theta}-\frac{4}{3}(2\tau_2+2\tau_3)}\Big)
\end{aligned}
$$

$$
\begin{aligned}
P(BABA) = \frac{3}{64}\Big(1 &- \frac{k}{(1+a)} e^{-\frac{8\tau_1}{3}} + \frac{3k-1}{(1+a)} e^{-\frac{8\tau_2}{3}} + \frac{2-k}{(1+a)} e^{-\frac{8\tau_3}{3}} - \frac{4k}{(1+a)(2+a)} e^{-\frac{4}{3}(\tau_1+2\tau_2)} \\
&- \frac{4k}{(1+a)(2+a)} e^{-\frac{4}{3}(\tau_1+2\tau_3)} - \frac{4(1+k)}{(1+a)(2+a)} e^{-\frac{4}{3}(\tau_2+2\tau_3)} + \frac{k}{(1+a)^2} e^{-\frac{4}{3}(2\tau_1+2\tau_3)} \\
&+ \frac{16k}{(1+a)(2+a)^2} e^{-\frac{4}{3}(\tau_1+\tau_2+2\tau_3)} + \frac{a(32+40a+10a^2)k}{(1+a)^2(2+a)^2(3+a)} e^{-\frac{2(\tau_2-\tau_1)}{\theta}-\frac{4}{3}(2\tau_2+2\tau_3)}\Big)
\end{aligned}
$$

$$
\begin{aligned}
\Rightarrow P(BBAA) - P(BABA) = \frac{3}{64}\Big(&\frac{4k}{(1+a)}\left(e^{-\frac{8\tau_1}{3}} - e^{-\frac{8\tau_2}{3}}\right) \\
&+ \frac{8k}{(1+a)^2} e^{-\frac{4}{3}(2\tau_1+2\tau_3)} - \frac{32k}{(1+a)(2+a)^2} e^{-\frac{4}{3}(\tau_1+\tau_2+2\tau_3)} \\
&- \frac{8ka^2}{(1+a)^2(2+a)^2} e^{-\frac{2(\tau_2-\tau_1)}{\theta}-\frac{4}{3}(2\tau_2+2\tau_3)}\Big)
\end{aligned}
$$

$\because \frac{4k}{(1+a)}\left(e^{-\frac{8\tau_1}{3}} - e^{-\frac{8\tau_2}{3}}\right) > 0$ and

$$
\begin{aligned}
&\frac{8k}{(1+a)^2} e^{-\frac{4}{3}(2\tau_1+2\tau_3)} - \frac{32k}{(1+a)(2+a)^2} e^{-\frac{4}{3}(\tau_1+\tau_2+2\tau_3)} \\
&\quad > \left(\frac{8k}{(1+a)^2} - \frac{32k}{(1+a)(2+a)^2}\right) e^{-\frac{4}{3}(\tau_1+\tau_2+2\tau_3)} = \frac{8ka^2}{(1+a)^2(2+a)^2} e^{-\frac{4}{3}(\tau_1+\tau_2+2\tau_3)}
\end{aligned}
$$

$\therefore P(BBAA) - P(BABA) >$

$$
\frac{3}{64}\left(\frac{8ka^2}{(1+a)^2(2+a)^2} e^{-\frac{4}{3}(\tau_1+\tau_2+2\tau_3)} - \frac{8ka^2}{(1+a)^2(2+a)^2} e^{-\frac{2(\tau_2-\tau_1)}{\theta}-\frac{4}{3}(2\tau_2+2\tau_3)}\right) > 0
$$

Supplementary Note 4

In cases of $\gamma = 0$, when $\tau_1, \tau_2 \rightarrow 0$, and $\lambda\tau_1, \tau_3 \rightarrow \infty$, there is:

$$P(BABA) \rightarrow \frac{3}{64}(1 - \frac{1}{1 + 4/3\theta})$$

$$P(ABBA) \rightarrow \frac{3}{64}(1 + \frac{3}{1 + 4/3\theta})$$

$$\Rightarrow D = \frac{P(ABBA) - P(BABA)}{P(ABBA) + P(BABA)} \rightarrow \frac{4}{1 + 4/3\theta}/(2 + \frac{2}{1 + 4/3\theta})$$

$$if\ \theta \ll 1, D \rightarrow 1.$$

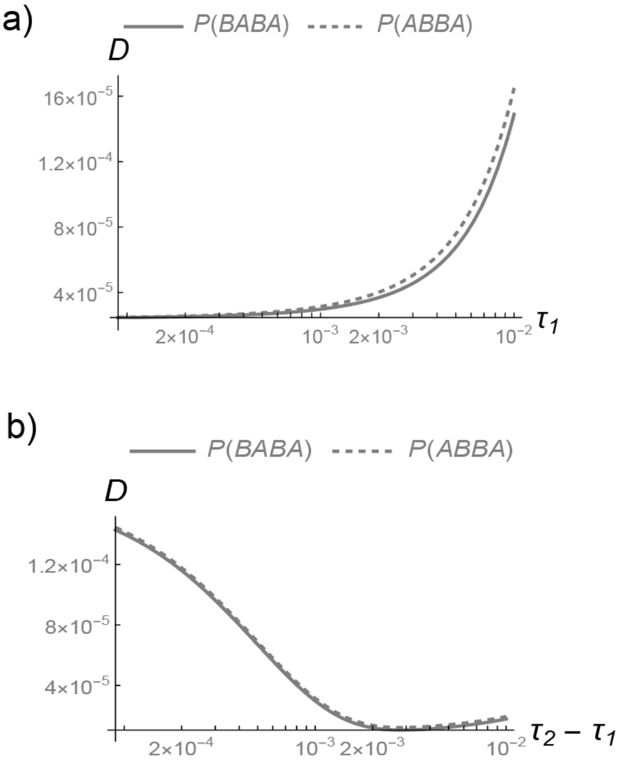

Figure S3. The frequencies of *ABBA* and *BABA* site patterns as τ_1 and $\tau_2 - \tau_1$ vary.

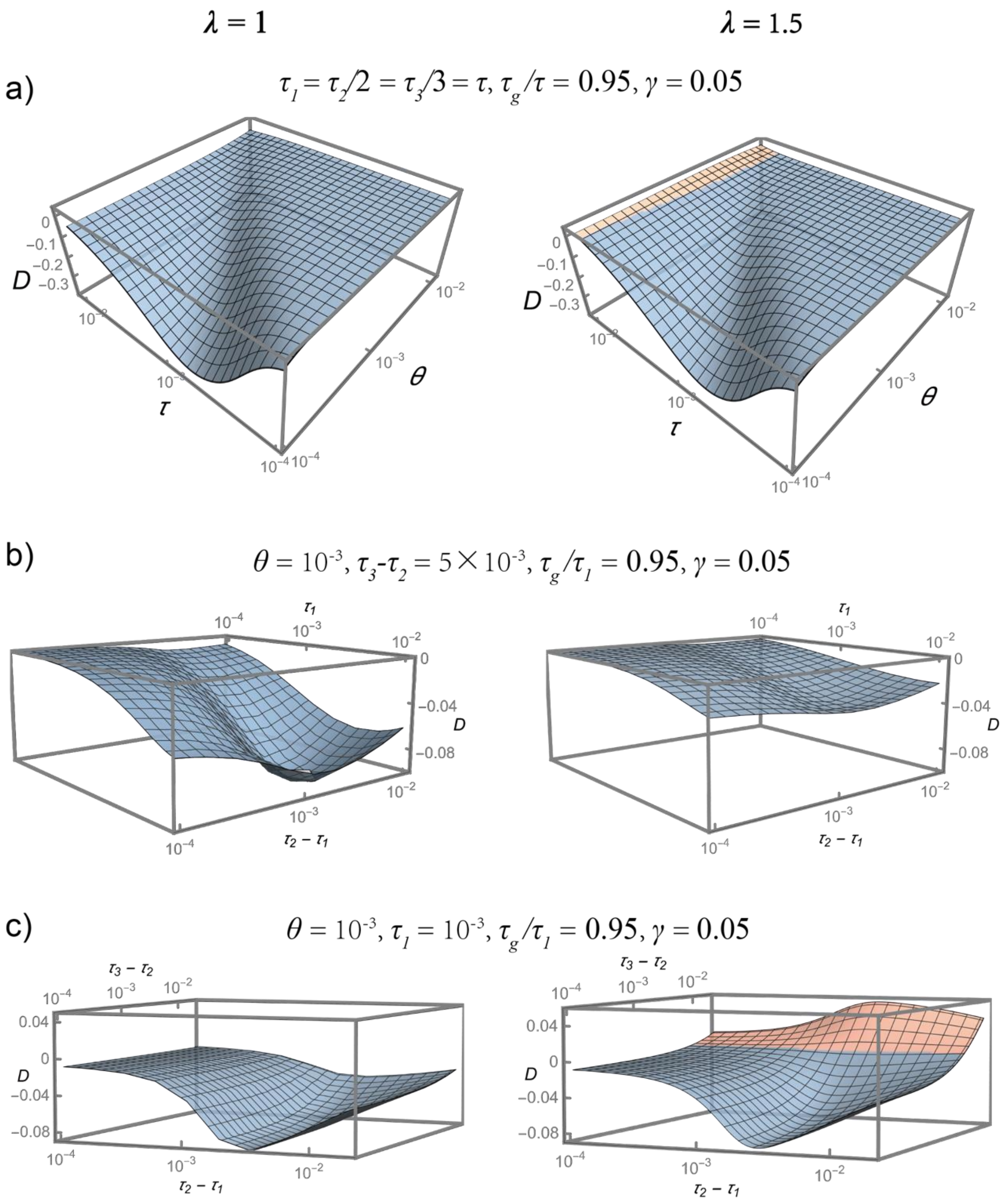

Figure S4. The effects of θ and the branch lengths τ_1, $\tau_2-\tau_1$ and $\tau_3-\tau_2$ on *D*-values in the presence of introgression.

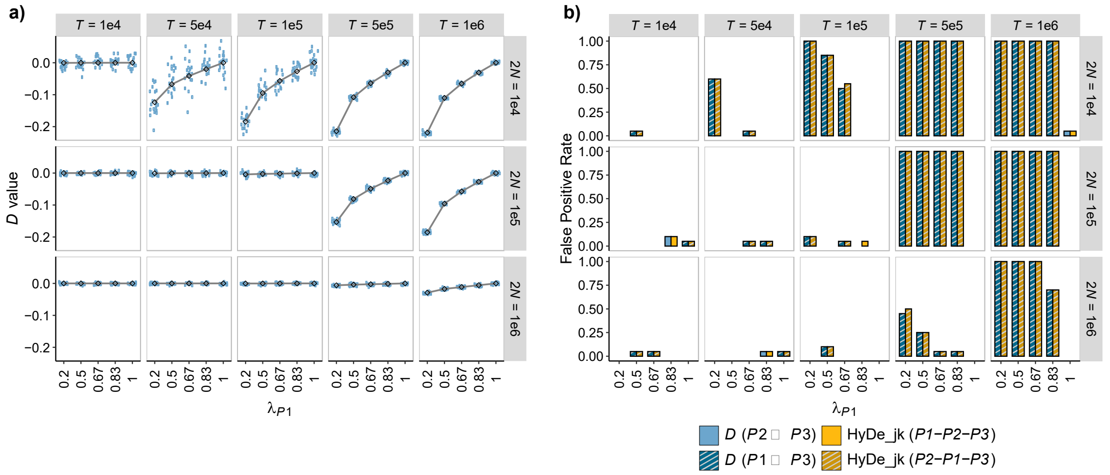

Figure S5. Interactive effects of phylogenetic depth (*T*), population size (2*N*), and rate variation on introgression testing. The simulated scenarios correspond to Figure 3a. The values on the strips at the top and right of each plot indicate the phylogenetic depth (*T*) and population size (2*N*), respectively. λ_{P1} is labeled on the x-axis. a) *D*-values: Colored points represent *D*-value estimates, with solid lines indicating the theoretical expected *D*-values. b) False-positive rate of *D*-statistic and HyDe_jk.

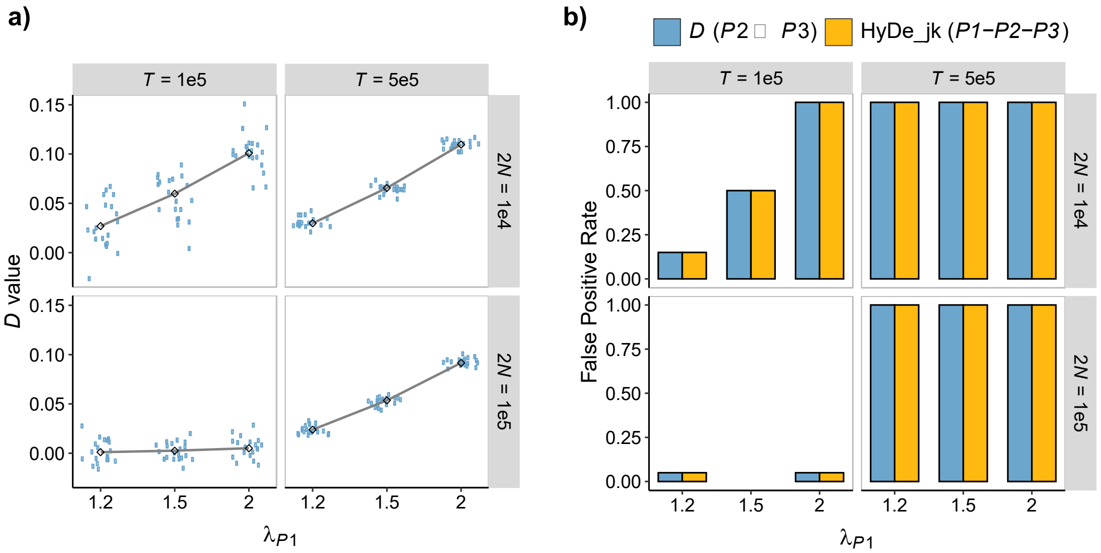

Figure S6. Performance comparison using 1000-bp datasets. The simulated scenarios correspond to Figure 3a but were generated using sequences of 1000 bp in length. The results are compared to those in Figure 3b–c, which were based on 100-bp datasets.

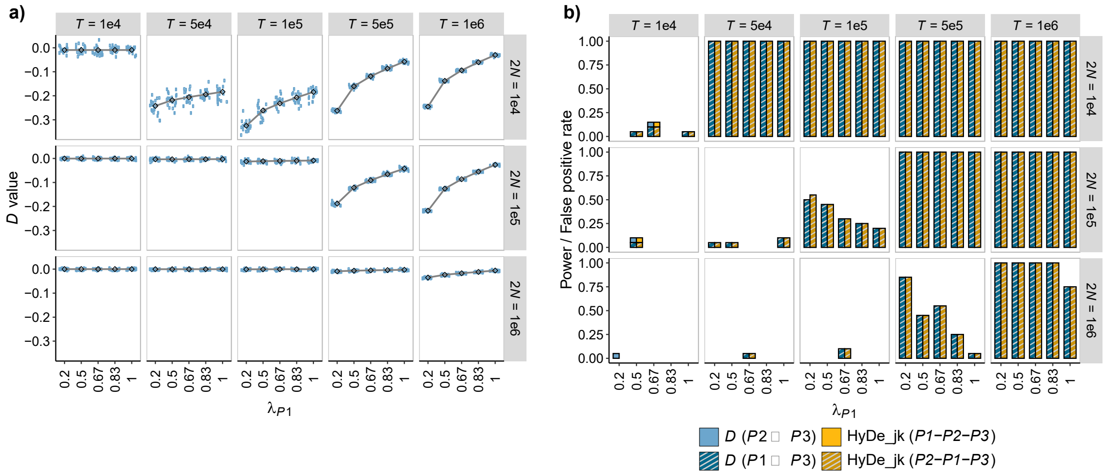

Figure S7. Effect of rate variation on performance of *D*-statistic and HyDe, in presence of introgression. The simulated scenarios correspond to Figure 7a. The values on the strips at the top and right of each plot indicate the phylogenetic depth (*T*) and population size (2*N*), respectively. λ_{P1} is labeled on the x-axis. a) *D*-values: Colored points represent *D*-value estimates, with solid lines indicating the theoretical expected *D*-values. b) False-positive rate of *D*-statistic and HyDe_jk.

Reference

Gardner EM, Bruun-Lund S, Niissalo M, Chantarasuwan B, Clement WL, Geri C, Harrison RD, Hipp AL, Holvoet M, Khew G. 2023. Echoes of ancient introgression punctuate stable genomic lineages in the evolution of figs. Proc. Natl. Acad. Sci. U.S.A. 120:e2222035120.

Graur D, Li W-HL. 2000. Fundamentals of molecular evolution. 12th ed. Sunderland, MA: Sinauer.

Karimi N, Grover CE, Gallagher JP, Wendel JF, Ané C, Baum DA. 2020. Reticulate evolution helps explain apparent homoplasy in floral biology and pollination in baobabs (*Adansonia*; Bombacoideae; Malvaceae). Syst. Biol. 69:462–478.

Leduc-Robert G, Maddison WP. 2018. Phylogeny with introgression in *Habronattus* jumping spiders (Araneae: Salticidae). BMC Evol. Biol. 18:1–23.

Liu BB, Ren C, Kwak M, Hodel RG, Xu C, He J, Zhou WB, Huang CH, Ma H, Qian GZ. 2022. Phylogenomic conflict analyses in the apple genus *Malus* sl reveal widespread hybridization and allopolyploidy driving diversification, with insights into the complex biogeographic history in the Northern Hemisphere. J. Integr. Plant Biol. 64:1020–1043.

Meng C, Kubatko LS. 2009. Detecting hybrid speciation in the presence of incomplete lineage sorting using gene tree incongruence: A model. Theor. Popul. Biol. 75:35–45.

Rannala B, Yang Z. 2003. Bayes estimation of species divergence times and ancestral population sizes using DNA sequences from multiple loci. Genetics 164:1645–1656.

Richards A, Kubatko L. 2022. Site pattern probabilities under the multispecies coalescent and a relaxed molecular clock: Theory and applications. J. Theor. Biol. 542:111078.

Sarver BA, Herrera ND, Sneddon D, Hunter SS, Settles ML, Kronenberg Z, Demboski JR, Good JM, Sullivan J. 2021. Diversification, introgression, and rampant cytonuclear discordance in rocky mountains chipmunks (Sciuridae: *Tamias*). Syst. Biol. 70:908–921.

Wu M, Kostyun JL, Hahn MW, Moyle LC. 2018. Dissecting the basis of novel trait evolution in a radiation with widespread phylogenetic discordance. Mol. Ecol. 27:3301–3316.

Zhu S, Degnan JH. 2017. Displayed trees do not determine distinguishability under the network multispecies coalescent. Syst. Biol. 66:283–298.
